# BMP Signaling Modulates *Hepcidin* Expression in Zebrafish Embryos Independent of *Hemojuvelin*

**DOI:** 10.1371/journal.pone.0014553

**Published:** 2011-01-21

**Authors:** Yann Gibert, Victoria J. Lattanzi, Aileen W. Zhen, Lea Vedder, Frédéric Brunet, Sarah A. Faasse, Jodie L. Babitt, Herbert Y. Lin, Matthias Hammerschmidt, Paula G. Fraenkel

**Affiliations:** 1 Division of Hematology/Oncology, Beth Israel Deaconess Medical Center and Harvard Medical School, Boston, Massachusetts, United States of America; 2 Institut de Génomique Fonctionnelle de Lyon, Ecole Normale Supérieure, Lyon, France; 3 Program in Membrane Biology, Division of Nephrology, Massachusetts General Hospital, Boston, Massachusetts, United States of America; 4 Institute for Developmental Biology, University of Cologne, Koeln, Germany; Ecole Normale Supérieure de Lyon, France

## Abstract

Hemojuvelin (Hjv), a member of the repulsive-guidance molecule (RGM) family, upregulates transcription of the iron regulatory hormone *hepcidin* by activating the bone morphogenetic protein (BMP) signaling pathway in mammalian cells. Mammalian models have identified *furin*, *neogenin*, and *matriptase-2* as modifiers of Hjv's function. Using the zebrafish model, we evaluated the effects of *hjv* and its interacting proteins on *hepcidin* expression during embryonic development. We found that *hjv* is strongly expressed in the notochord and somites of the zebrafish embryo and that morpholino knockdown of *hjv* impaired the development of these structures. Knockdown of *hjv* or other *hjv*-related genes, including zebrafish orthologs of *furin* or *neogenin*, however, failed to decrease *hepcidin* expression relative to liver size. In contrast, overexpression of *bmp2b* or knockdown of *matriptase-2* enhanced the intensity and extent of *hepcidin* expression in zebrafish embryos, but this occurred in an *hjv*-independent manner. Furthermore, we demonstrated that zebrafish *hjv* can activate the human *hepcidin* promoter and enhance BMP responsive gene expression in vitro, but is expressed at low levels in the zebrafish embryonic liver. Taken together, these data support an alternative mechanism for *hepcidin* regulation during zebrafish embryonic development, which is independent of *hjv*.

## Introduction

Bone morphogenetic proteins (BMPs), originally identified for their ability to induce bone differentiation, are members of the TGF-β superfamily. Binding of a BMP molecule to a BMP receptor complex results in phosphorylation of Smad1, 5, and 8. These proteins then form hetero-oligomers with Smad4, translocate to the nucleus, and activate transcription of a target gene (reviewed in [1] and [2]). The proteins Chordin and Noggin antagonize BMP activity by binding BMPs and preventing their interaction with BMP receptors.

Hemojuvelin (Hjv, also known as RGMc), a protein belonging to the repulsive-guidance molecule (RGM) family, was originally identified as the affected gene in several families with severe early onset iron overload and reduced levels of hepcidin.[3] Hepcidin, a transcriptionally regulated peptide hormone, is produced in the liver[4] and modulates intestinal iron absorption and macrophage iron release[5]–[7]. The identification of Hjv linked the regulation of *hepcidin* expression and iron homeostasis to the BMP pathway. Subsequent studies revealed that membrane-bound Hjv binds Neogenin[8], increases intracellular iron accumulation[8], and enhances BMP-mediated induction of *hepcidin* expression in vitro[9], while Neogenin deficiency decreases hepatic hjv protein levels, impairs BMP signaling, and reduces *hepcidin* expression in postnatal mice.[10]

Although *hjv* expression is not iron responsive[8], [11], iron deficiency induces production of soluble hjv[8], while iron loading inhibits release of soluble hemojuvelin.[12], [13] It has been proposed that soluble Hjv, produced via a Furin-mediated proteolysis of membrane-bound Hjv[13], [14], antagonizes the function of membrane-bound Hjv[12], [15] resulting in low levels of *hepcidin* expression. Recently another membrane-bound cell surface serine protease, Matriptase-2 (Mtp2, also known as TMPRSS-6) has been shown to decrease *hepcidin* transcription[16] and to bind and cleave Hjv[17] in vitro.

We have been developing the zebrafish embryo (*Danio rerio*) as a model to study the developmental regulation of *hepcidin*. We have demonstrated that *hepcidin* expression begins at 36 hpf in the zebrafish embryo and that the zebrafish ortholog of Transferrin, *transferrin-a*, is required for *hepcidin* expression during embryonic development.[18] While the BMP pathway has been studied for its effect on embryonic symmetry and patterning,[19] its effect on *hepcidin* regulation during embryonic development has not been characterized previously. Furthermore, the effects of *hjv* and related genes on *hepcidin* expression have not been evaluated previously during embryonic development.

In this report, we demonstrate that activation of the BMP pathway increased the intensity and extent of hepatic *hepcidin* expression during embryonic development, and suppression of BMP signaling by the chemical inhibitor dorsomorphin eliminated *hepcidin* expression. In contrast, knockdown of *hjv* reduced the size of the liver, but failed to eliminate *hepcidin* expression. While knockdown of *mtp2* increased *hepcidin* expression, relative to liver size, this effect was independent of *hjv*. As experimental overexpression of *hjv* in zebrafish embryos failed to increase *hepcidin* expression, we propose that the regulation of *hepcidin* expression in zebrafish embryos is *hjv*-independent.

## Results

### Induction of *bmp2b* at 48 hpf stimulates *hepcidin* expression in zebrafish embryos

**Figure 1:**
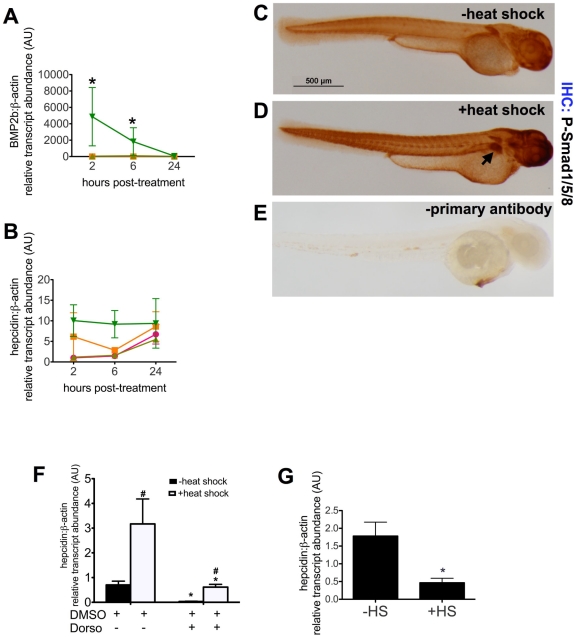
The BMP pathway regulates *hepcidin* expression in zebrafish embryos. A–B. Time course of *bmp2b* and *hepcidin* expression following induction of *BMP2b* expression. Tg(*hsp70:bmp2b*) is a transgenic line of zebrafish, which carries the *BMP2b* gene under the control of the *hsp70* promoter. At 48 hours post-fertilization (hpf), WT or tg(*hsp70:bmp2b*) groups of embryos (n = 20 embryos per group) were subjected either to heat shock (+HS) at 37°C for 40 min or maintained at the usual temperature (28°C) (−HS). Pools of embryos were obtained for RNA extraction at 2, 6, and 24 hours after the start of heat shock, corresponding to 50, 54, and 72 hpf. Quantitative real-time RT-PCR was performed to measure transcript levels of *bmp2b* (**A**) or *hepcidin* (**B**), normalized to *β-actin* transcript levels and measured as fold increase over control, WT,−HS at 2 hours post-treatment. Data shown are means ± SE. * indicates p<0.05, compared to control. N = 2 pools per group. WT, −HS (pink circles), WT, +HS (orange squares), transgenic, −HS (light green triangles), transgenic, +HS (dark green triangles). **C–E.** Immunohistochemistry for P-Smad1/5/8. Compared to zebrafish embryos without *BMP2b* induction (**C**), P-Smad1/5/8 staining is increased in the liver (arrow) in tg(*hsp70*:*BMP2b*) embryos following heat shock (**D**). Omitting the primary antibody (anti-P-smad1/5/8), but including the biotinylated anti-Rabbit IgG/streptavidin horseradish peroxidase resulted in very low levels of background staining (**E**). N = 20 embryos per group. **F,G.** Inhibition of *hepcidin* expression by dorsomorphin (**F**) or *noggin3* (**G**). **F.** From 28–55 hpf, pools of tg(*hsp70:BMP2b*) embryos were treated with the BMP inhibitor, 40 µM dorsomorphin (+Dorso), or treated with an equivalent amount of DMSO vehicle alone (+DMSO). Half the pools of embryos were subjected to heat shock at 48 hpf to induce *bmp2b* expression, followed by fixation at 55 hpf for quantitative real-time RT-PCR. **G.** Pools of embryos carrying tg(*hsp70*:*noggin3*) were subjected to heat shock or no heat shock at 48 hpf. The embryos were fixed at 55 hpf for quantitative real-time RT-PCR. Data shown are means ± SE. N = 4–5 pools per group. * indicates p<0.05, compared with no heat shock and no dorsomorphin treatment. # indicates p<0.05 compared with previous column.

BMP signaling has been shown to modulate *hepcidin* expression in adult mammals[9], [20] and in adult zebrafish.[21] As BMP2 has been demonstrated to stimulate *hepcidin* transcription in mammalian cell culture[20], we exploited the tg(*hsp70:bmp2b*) line of zebrafish[19] to assess whether BMP signaling regulates *hepcidin* expression in the zebrafish embryo. Tg(*hsp70:bmp2b*) transgenic zebrafish carry the *bmp2b* gene, one of two zebrafish orthologs of BMP2, under the control of the *hsp70* promoter. Transgenic animals were incrossed to generate embryos, which were subjected to heat shock, or no heat shock, at 48 hours post-fertilization. Pools of embryos were harvested at 2, 6, and 24 hours post-treatment and assayed for *bmp2b* expression (Figure 1A) in comparison to nontransgenic embryos at 48 hpf, which were not subjected to heat shock. Quantitative real-time RT-PCR revealed a 2000-fold increase in *bmp2b* expression in the transgenic embryos two hours after heat shock compared to nontransgenic embryos subjected to heat shock (4866±3556 vs 1.12±0.184, p<0.001) or 100-fold increase compared to transgenic embryos not subjected to heat shock (4866±3556 vs 44.85±14.85, p<0.01). In the transgenic embryos, *bmp2b* expression remained significantly elevated 6 hours after heat shock, but 24 hours after heat shock *bmp2b* expression declined to non heat-shock levels of expression (58.8±12.2).

Induction of *hepcidin* expression corresponded with induction of *bmp2b* expression in the tg(*hsp70*:*bmp2b*) embryos. Two hours after the start of the heat shock, *hepcidin* expression levels increased ten-fold (Figure 1B), compared to untreated WT embryos (10.1±3.86 vs 1.00±0.03) or compared to untreated transgenic embryos (10.1±3.86 vs 1.15±0.398). As elevations in *hepcidin* expression persisted in the transgenic embryos at 6 hours after the start of heat shock (54 hpf) and were not associated with an increase in *hepcidin* expression in WT embryos subjected to heat shock, this time point was selected for subsequent experiments. At 24 hours post heat shock, or 72 hpf, *hepcidin* transcript levels increased in both the heat shock and non heat shock treated WT and non heat shock treated transgenic embryos. This is consistent with a developmental increase in *hepcidin* expression from 54 to 72 hpf, which we have observed previously[18]. To confirm that heat shock activated the BMP signaling pathway in tg(*hsp70:bmp2b*) embryos, we performed whole mount immunohistochemistry (Figure 1C–E) for phosphorylated Smad1, 5, and 8 proteins, which revealed increased staining for these phosphoproteins in the liver, somites, and head 6 hours after heat shock (55 hpf).

### Inhibition of BMP type I receptors decreases *hepcidin* expression in zebrafish embryos

To evaluate further the role of BMP signaling in *hepcidin* regulation during embryogenesis we selectively inhibited BMP type I receptors using the recently identified BMP signaling inhibitor, dorsomorphin.[21] Dorsomorphin was previously shown to dorsalize embryos, expanding structures derived from the dorsal pole, when added before 12 hpf.[21] By delaying the addition of dorsomorphin until 28 hpf, and then maintaining them in the chemical until 55 hpf, we found that the embryos exhibited normal embryonic patterning, but exhibited a dose-dependent decrease in *hepcidin* expression by quantitative realtime RT-PCR from 1 to 40 µM (data not shown). We chose to use 40 µM dorsomorphin, which produced near complete inhibition of *hepcidin* expression. We then incubated pools of tg(*hsp70*:*bmp2b*) embryos in 40 µM dorsomorphin/0.3% DMSO or in 0.3% DMSO alone from 28–55 hpf. Half the pools were subjected to heat shock at 48 hpf to induce *bmp2b* expression. The embryos were fixed at 55 hpf for quantitative real-time RT-PCR. In the absence of dorsomorphin (Figure 1F), heat shock significantly increased *hepcidin* expression (3.17±1.01 vs 0.702±0.154, p<0.01). In the absence of heat shock, dorsomorphin exposure reduced *hepcidin* expression 20-fold (0.032±0.012 vs 0.702± 0.154, p<0.001). In the presence of heat shock, dorsomorphin diminished the effect of *bmp2b* induction on *hepcidin* expression six-fold, but failed to abrogate it. Immunohistochemical staining demonstrated that dorsomorphin decreased, but did not eliminate phospho-smad1,5,8 staining, in transgenic embryos treated with heat shock (Figure S1). Thus it appears that the 2000-fold increase observed in BMP2b expression following heat shock of transgenic embryos partially overcomes the inhibitory effects of dorsomorphin on BMP signaling.

We found further support that BMP signaling regulates *hepcidin* expression in zebrafish embryos, by using transgenic zebrafish that express the BMP signaling antagonist *noggin3* under the control of the *hsp70* promoter [19]. We crossed these tg(*hsp70*:*noggin3*) zebrafish to WT fish producing progeny in which 50% of the embryos carried the transgene. We then heat shocked these progeny at 48 hpf and fixed at 55 hpf for quantitative realtime RT-PCR. Heat shocked embryos exhibited a significant reduction (Figure 1G) in the *hepcidin* transcript levels (1.78±0.39 vs. 0.467±0.126, p = 0.027), consistent with inhibition of *hepcidin* expression by *noggin3*.

### Knockdown of *hjv* causes notochord and somite defects

**Figure 2:**
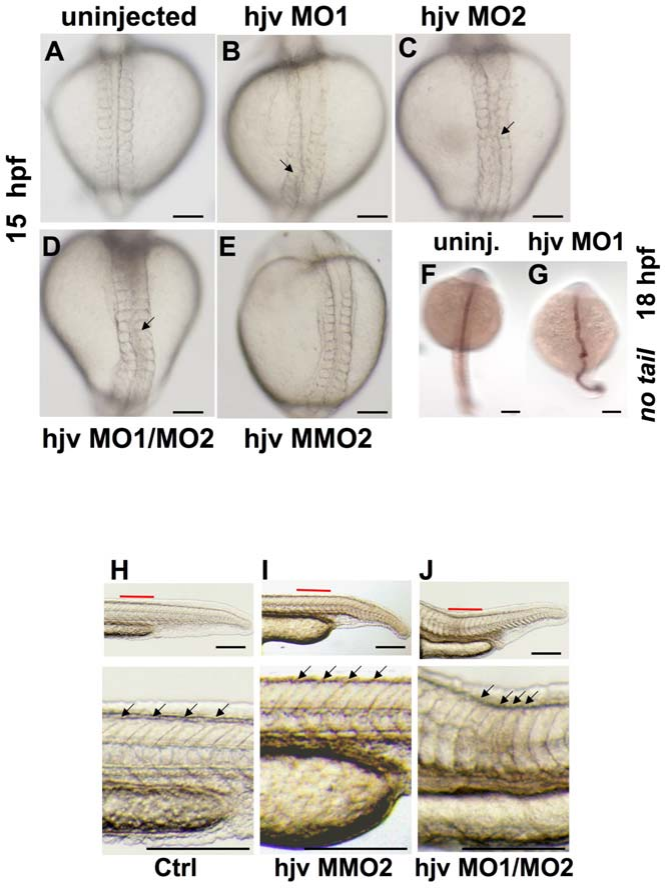
Morpholino knockdown of *hjv* results in notochord and somite abnormalities. **A–E.** Light microscopy of zebrafish embryos at 15 hpf in dorsal view Compared to uninjected embryos (**A**) or embryos injected with a mismatch control morpholino (**E**), embryos injected with either single hjv morpholinos (**B, C**) or a combination of hjv MO1 and hjv MO2 (**D**) exhibited a distorted notochord (arrows). N = 30 per group. **F,G.** Whole mount *in situ* hybridization at 18 hpf with *no tail*, which stains the notochord, illustrates the bent shape in the hjv MO1 injected morphants. N = 15 per group. **H–J.** Light microscopy of the tail at 24 hpf lateral view (top) with additional 3.5x enlargement of area labeled in red (below). Somites (arrows) in uninjected (**H**) and control morpholino-injected embryos (**I**) appeared V-shaped, while somites appeared U-shaped with decreased anterior-posterior dimension (distance between each pair of arrows) in *hjv* morphants (**J**). N = 20 per group.

To assess whether *hjv* is required to induce *hepcidin* expression during zebrafish embryogenesis, we injected antisense morpholinos (MOs) at the one-cell stage to knock down the *hjv* gene. Hjv MO1 targets the 5′ UTR of *hjv* and is designed to impair translation of *hjv*, while hjv MO2 is a non-overlapping morpholino targeting the second exon donor site of the coding sequence. Injection of either morpholino at 0.5 mM was associated with severe growth retardation, which impaired the ability of the embryo to develop past 18 hpf (data not shown) to the expected time of onset of *hepcidin* expression[18] (36 hpf). At a lower injection concentration, 0.2 mM, injected embryos were able to develop past somitogenesis. Compared to uninjected embryos (Figure 2A,F), embryos injected with either hjv MO1 (Figure 2B,G) or hjv MO2, (Figure 2C) exhibited undulating notochord and body axis at 15–18 hpf, visible on light microscopy or by whole mount *in situ* hybridization for the notochord specific marker, *no tail* (Figure 2F,G). Co-injecting hjv MO1 and hjv MO2 exacerbated notochord distortion (Figure 2D), however injection of a mismatch control morpholino (hjv MMO2) did not distort the notochord (Figure 2E). While zebrafish embryonic somites exhibited a well-delineated V-shape at 24 hpf in uninjected or control morpholino injected embryos (Figure 2H,I), the somites were decreased in the anterior-posterior dimension and U-shaped in *hjv* morphants (Figure 2J).

### Induction of *bmp2b* increases the intensity and extent of *hepcidin* expression without affecting liver size

**Figure 3:**
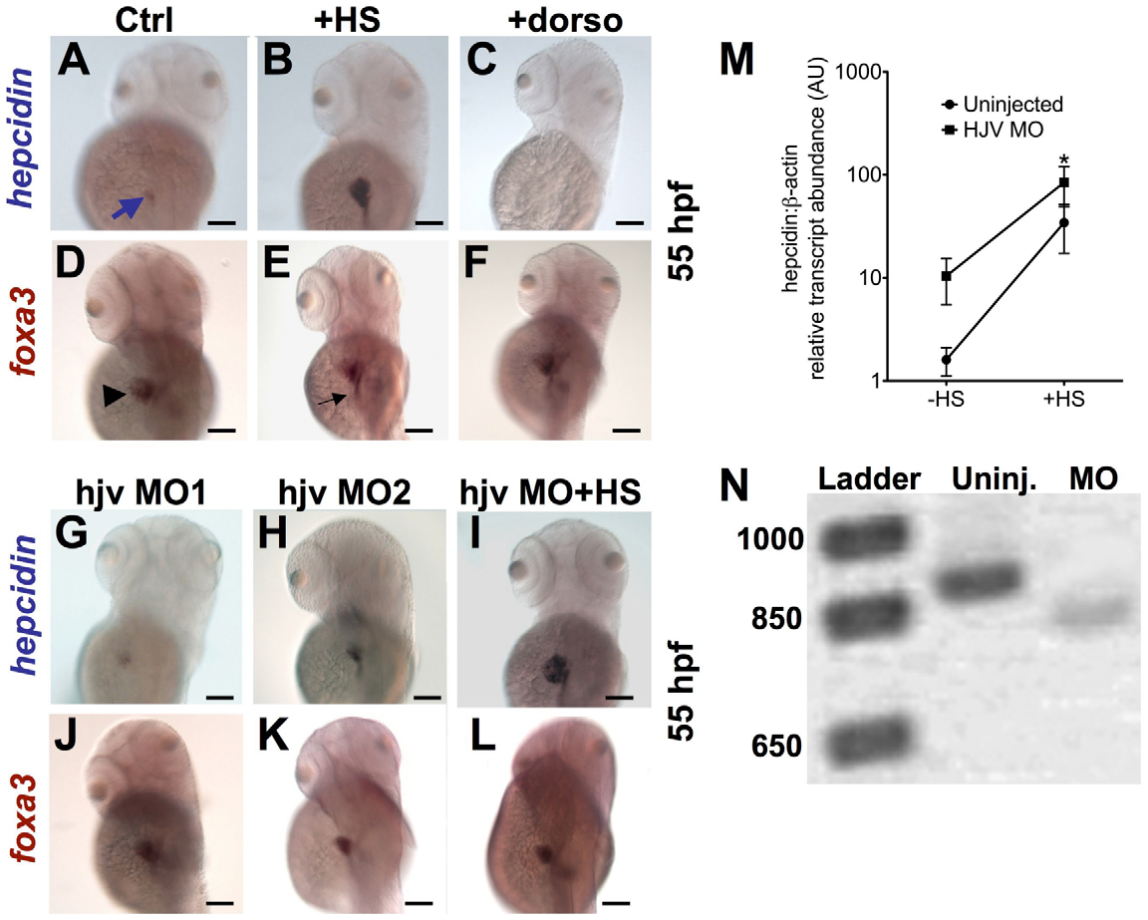
Knockdown of *hjv* does not significantly impair *hepcidin* expression at 55 hpf. **A–L.** Whole mount *in situ* hybridization at 55 hpf for *hepcidin* (blue arrow) (**A–C**, **G–I**) and *foxa3* (**D–F**, **J–L**), as a marker for the liver (arrowhead) and intestine (black arrow). Compared to controls (**A**,**D**), induction of *bmp2b* by heat shock in tg(*hsp70*: *bmp2b*) embryos (**B**,**E**) increased *hepcidin* expression. Treatment with dorsomorphin from 28–55 hpf in WT embryos abrogated *hepcidin* expression, without affecting liver size (**C**,**F**). Knockdown of *hjv* by a morpholino blocking translation (**G**,**J**), or by a non-overlapping morpholino targeting a splice acceptor site (**H**,**K**), did not significantly change *hepcidin* expression, but slightly reduced liver size. Knockdown of *hjv* in tg(*hsp70:bmp2b*) embryos failed to prevent strong *hepcidin* expression following induction of *bmp2b* (**I**,**L**). N = 10–30 embryos per group. **M.** The effect of *hjv* knockdown on *bmp2b*-induced *hepcidin* transcript levels assessed by quantitative realtime RT-PCR. Embryos were injected with hjv MO2 at the one-cell stage followed by heat shock (HS) at 48 hpf and fixation for RNA extraction at 55 hpf. **N.** Electrophoresis of RT-PCR products, which were designed to amplify the targeted splice site, confirmed an 80 basepair alteration in transcript size, consistent with aberrant splicing of the *hjv* transcript in the morphants.

As Hjv has been shown to function as a BMP co-receptor in mammalian models, we assessed the effect of BMP signaling and *hjv* on *hepcidin* expression. In comparison to uninjected WT embryos at 55 hpf (Figure 3A), induction of *bmp2b* by heat shock at 48 hpf in tg(*hsp70:bmp2b*) resulted in increased intensity and extent of *hepcidin* expression in the liver and foregut (Figure 3B) by whole mount *in situ* hybridization, while treatment with dorsomorphin from 28–55 hpf (Figure 3C) abrogated *hepcidin* expression. Transgenic induction of the BMP antagonist *noggin3* at 48 hpf produced an equivalent effect (data not shown). While early BMP signaling is important for embryonic liver development[22], induction of *bmp2b* at 48 hpf, which is after specification of the liver, did not increase liver size (Figure 3D,E), as assessed by whole mount *in situ* hybridization for *foxa3* (*forkhead box a3*), a gene expressed in zebrafish embryonic liver tissue[23]. Treatment with the BMP signaling inhibitor dorsomorphin from 28–55 hpf, also failed to decrease liver size (Figure 3F).

### Knockdown of *hjv* fails to impair *hepcidin* expression at 55 hpf

To evaluate whether *hjv* is required for *hepcidin* expression, we injected hjv MO1 or hjv MO2, and assessed *hepcidin* expression by *in situ* hybridization. Compared to uninjected controls (Figure 3A), neither hjv MO1 nor hjv MO2 (Figure 3G,H) exhibited decreased *hepcidin* expression, although the liver was slightly reduced in size (Figure 3J,K). As knockdown of *hjv* produced developmental defects, we evaluated the effects of *hjv* deficiency on hemoglobin production and found that *hjv* knockdown did not produce anemia (Figure S2). To test whether *hjv* is required for the stimulatory effect of *bmp2b* on *hepcidin* expression, we injected hjv MO2 in tg(*hsp70*:*bmp2b*) embryos at the one cell stage, followed by heat shock at 48 hpf and fixation at 55 hpf. Induction of *bmp2b* still enhanced *hepcidin* expression, despite knockdown of *hjv* (Figure 3I). Quantitative realtime RT-PCR (Figure 3M) revealed an 8-fold increase in *hepcidin* transcript levels (84.31±35.49 vs 10.41±4.93, p = 0.029) in *hjv* morphants following heat shock compared to morphants without heat shock. To confirm that the *hjv* gene was effectively knocked down in the *hjv* zebrafish morphant embryos, we extracted RNA from hjv-MO2 injected embryos and amplified the predicted splice site by RT-PCR. We found that the amplified region in the *hjv* morphants was shorter than in uninjected controls (Figure 3N). We cloned and sequenced the amplified product from the morphants and uninjected controls and confirmed that the morphant transcript bypasses the exon donor targeted by hjv-MO2 in favor of an aberrant splice from nucleotide 25 to 104 of the coding sequence. The predicted translation of this aberrant spliceform lacks amino acids 9 through 35, which is the majority of the signal peptide, as predicted by the algorithm PrediSi[24].

*Neogenin* and *furin* have been shown to interact with *hjv* to regulate *hepcidin* transcription in mammalian models.[8], [14] In zebrafish embryos, *neogenin* has previously been shown to be required for normal somite development[25], while the two zebrafish furins, *furina* and *furinb* participate in pharyngeal cartilage development.[26] We generated knockdowns of *neogenin* or of both *furina* and *furinb*, which did not exhibit impaired *hepcidin* expression or abnormal liver size at 55 hpf (Figure S3), although these knock downs reproduced the published developmental phenotypes (Figures S4 and S5).

### *Hjv* is weakly expressed in the zebrafish embryonic liver

**Figure 4:**
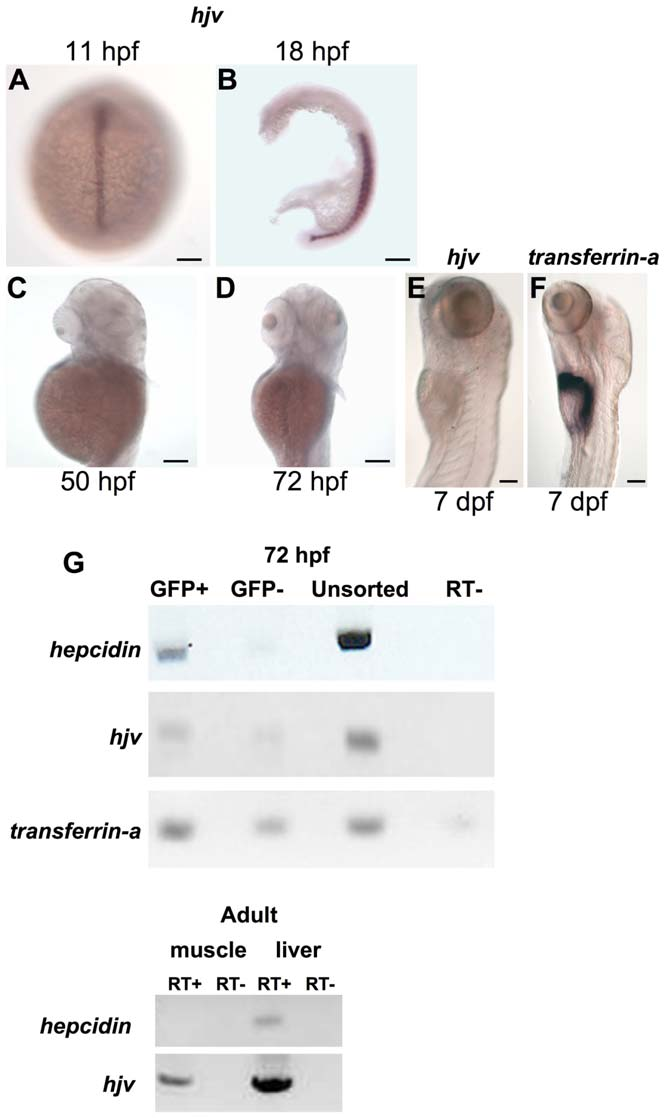
The *hjv* transcript is weakly expressed in the zebrafish embryonic liver at the time of *hepcidin* expression. **A–F.** Whole mount *in situ* hybridization for *hjv* (**A–E**) or *transferrin-a* (**F**) demonstrating expression in the notochord at 11 hpf (**A**) (dorsal view), in the somites at 18 hpf (**B**) (lateral view with yolk removed), but absence from the liver at 50 hpf (**C**), 72 hpf (**D**), and 7 days post-fertilization (dpf) (**E**). In comparison, *transferrin-a* is strongly expressed in the liver at 7 dpf (**F**). N = 10–30 embryos per group. **G.** Semiquantitative RT-PCR for *hepcidin*, *hjv*, and *transferrin-a* expression in embryonic zebrafish hepatocytes (top, GFP+) and nonhepatocytes (top, GFP-) and for *hepcidin* and *hjv* in adult zebrafish skeletal muscle and liver (bottom). Embryonic hepatocytes were sorted by FACS from pools of 80–100 transgenic zebrafish embryos at 72 hpf, which express GFP under the control of the liver-specific *LFABP* promoter. RT- indicates control reaction with reverse transcriptase omitted.

To determine why knockdown of *hjv* or related genes did not impair *hepcidin* expression, we evaluated a time course of *hjv* expression in zebrafish embryos by whole mount *in situ* hybridization. As previously reported,[27] we found that *hjv* is strongly expressed in the notochord at 11 hpf (Figure 4A) and in the developing somites at 18 hpf (Figure 4B), prior to the onset of liver development. We discovered that *hjv* was not detectable by *in situ* hybridization at 50 hpf, 72 hpf, or 7 days post-fertilization (dpf) (Figure 4C–E), in contrast to *hepcidin* (Figure 3) or *transferrin-a* ([18] and Figure 4F), which are evident in the liver.

By bioinformatic analysis, we identified three other members of the repulsive guidance molecule family in the zebrafish, *RGMa*, *RGMb*, and *RGMd*. We found that none of these paralogs of *hjv* were expressed in the zebrafish embryonic liver (Figure S6). Knockdown of each of them failed to impair *hepcidin* expression at 55 hpf (Figure S7). At 72 hpf (Figure S8), *hepcidin* transcript levels were normal in the *RGM* morphants, although liver development was impaired in *RGMb* and *RGMd* morphants.

To verify whether there was weak *hjv* expression in the liver during embryogenesis, which was undetected by *in situ* hybridization, we used a fluorescence activated cell sorter to sort hepatocytes from transgenic embryos, which expressed GFP under the control of the liver specific *liver fatty acid binding protein* (LFABP) promoter. RNA was obtained from sorted (GFP+ and GFP−) and from unsorted cells for RT-PCR. GFP+ cells strongly expressed *LFABP*, relative to *β-actin* (Figure S9A). In the sorted cells, *hjv* expression was below the detection level in a quantitative real-time PCR assay. We performed semi-quantitative RT-PCR, which revealed weak expression of *hjv* in both GFP+ and GFP− cells (Figure 4G). Comparing GFP+ to unsorted cells, the *hjv* expression was diminished in a similar proportion to that for the *hepcidin* transcript, and is thus consistent with hepatic expression, although at low levels. In contrast, *hepcidin* expression was evident only in the GFP+ cells and *transferrin-a* was detectable in both populations. In adult zebrafish, *hjv* transcripts were detected by RT-PCR in both skeletal muscle and liver (Figure 4G), similar to the adult human *hjv* expression pattern.[3] We also found that *neogenin* and the zebrafish paralogs of *hjv* were expressed in the adult zebrafish liver (Figure S9B). These data indicate that, hemojuvelin is a developmentally regulated gene, which exhibits low levels of expression in zebrafish embryonic hepatocytes, consistent with the *hjv*-independent regulation of *hepcidin* that we observed in the zebrafish embryo (Figure 3).

### Overexpression of zebrafish *hjv* fails to increase *hepcidin* expression in zebrafish embryos

**Figure 5:**
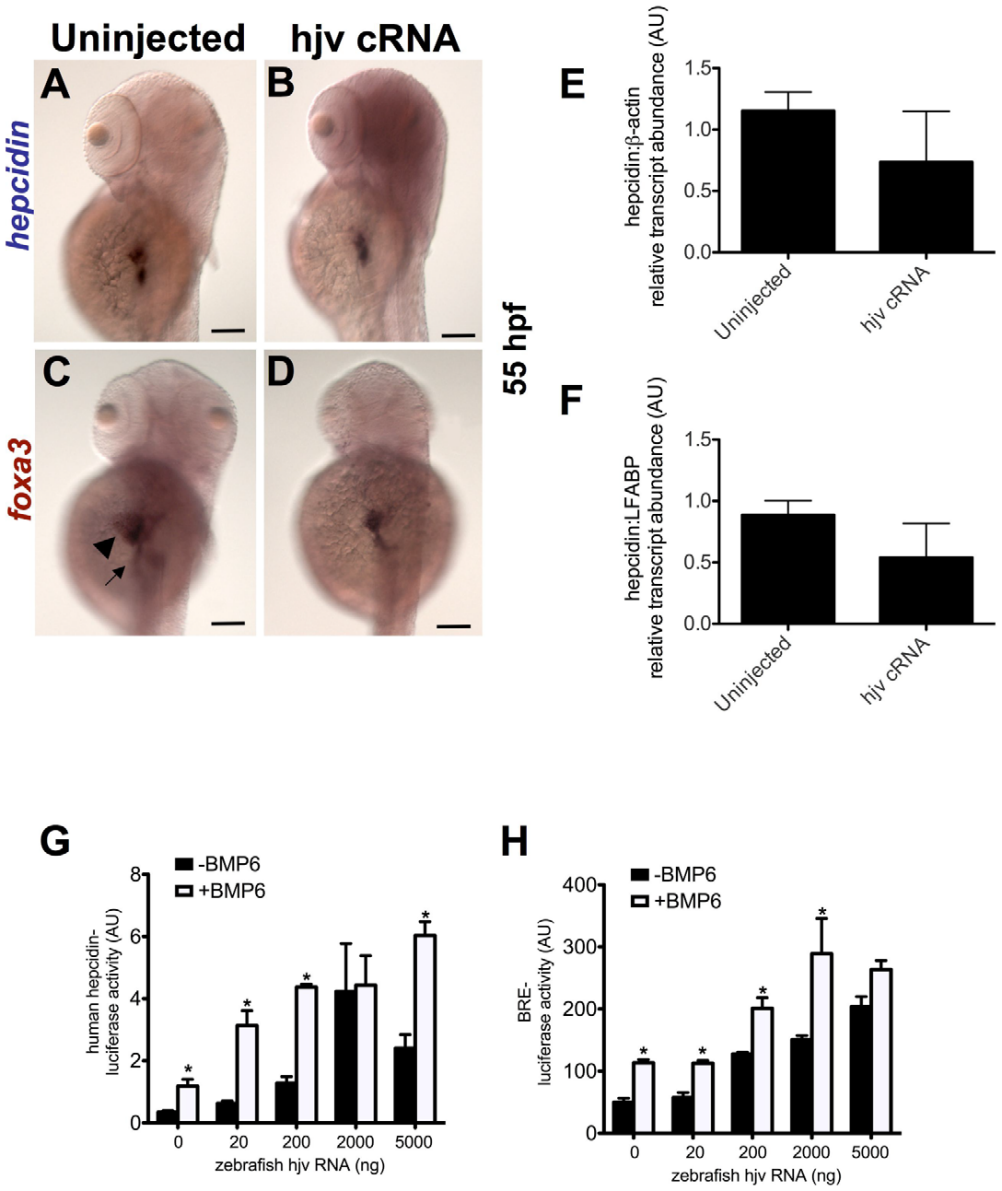
Overexpression of zebrafish *hjv* fails to increase *hepcidin* expression in zebrafish embryos, but can cooperate with BMP6 to activate the human *hepcidin* promoter *in vitro*. **A–D.** Whole mount *in situ* hybridization for *hepcidin* (**A**,**B**) or *foxa3* (**C**,**D**) as a marker for the liver (arrowhead) and intestine (arrow) at 55 hpf following injection of zebrafish *hjv* cRNA at the one cell stage. N = 20–30 embryos per group. **E**,**F.** Quantitative real-time RT-PCR at 72 hpf demonstrated no significant change in *hepcidin* expression relative to *β-actin* (**E**) or to *LFABP* (**F**) following overexpression of *hjv* cRNA. N = 2 pools per group. **G**,**H**. *In vitro* luciferase reporter assays in Hep3B cells demonstrate the effect of increasing doses of zebrafish *hjv* cRNA on the human *hepcidin* promoter (**G**) or the BMP response element (**H**) in the absence (black) or presence (white) of exogenous BMP6 (5 ng/ml). Relative light units were calculated as ratios of Firefly (reporter) and Renilla (transfection control) values. Results from luciferase assay experiments were expressed as the means ± standard error of triplicates from representative experiments. * denotes p<0.05, compared to the previous column.

To test the hypothesis that zebrafish *hjv* fails to regulate *hepcidin* expression during embryonic development because it is only weakly expressed in the embryonic liver, we injected zebrafish *hjv* cRNA at the one-cell stage and assessed *hepcidin* and *foxa3* expression at 55 hpf. Compared to uninjected embryos, *hjv* overexpression failed to increase *hepcidin* expression (Figure 5A–D). Quantitative real-time RT-PCR for *hepcidin* expression at 72 hpf normalized to *β-actin* (Figure 5E) or to *LFABP* (Figure 5F) failed to show an increase in *hepcidin* expression in embryos injected with *hjv* cRNA. To overcome concerns about potential degradation of the cRNA during development, we also injected at the one-cell stage a DNA construct (pHjv-CS2) containing the zebrafish *hjv* gene in the pCS2 vector under the control of a ubiquitous promoter. Similar to the results in Figure 5E, we found no significant increase in *hepcidin* expression in the transgenic embryos compared to embryos injected with the pCS2 vector alone (Figure S10).

### Zebrafish *hjv* induces *hepcidin* expression in human hepatocytes

As overexpression of zebrafish *hjv* failed to increase *hepcidin* expression in the zebrafish embryos, we questioned whether zebrafish *hjv* functions as a BMP co-receptor. To evaluate this, we cotransfected human hepatocytes (Hep3B cells) with increasing doses of zebrafish *hjv* cRNA and a reporter construct containing the human *hepcidin* promoter upstream of Firefly luciferase. Increasing doses of zebrafish *hjv* were associated with stronger induction of the human *hepcidin* promoter, which was potentiated by the addition of BMP6 (Figure 5G). Similarly, cotransfection of zebrafish *hjv* cRNA with a reporter construct containing a BMP response element upstream of luciferase, revealed a dose dependent increase in promoter activity, which was enhanced by the addition of BMP6 (Figure 5H).

### Knockdown of *matriptase-2* increases *hepcidin* expression in a BMP dependent manner

**Figure 6:**
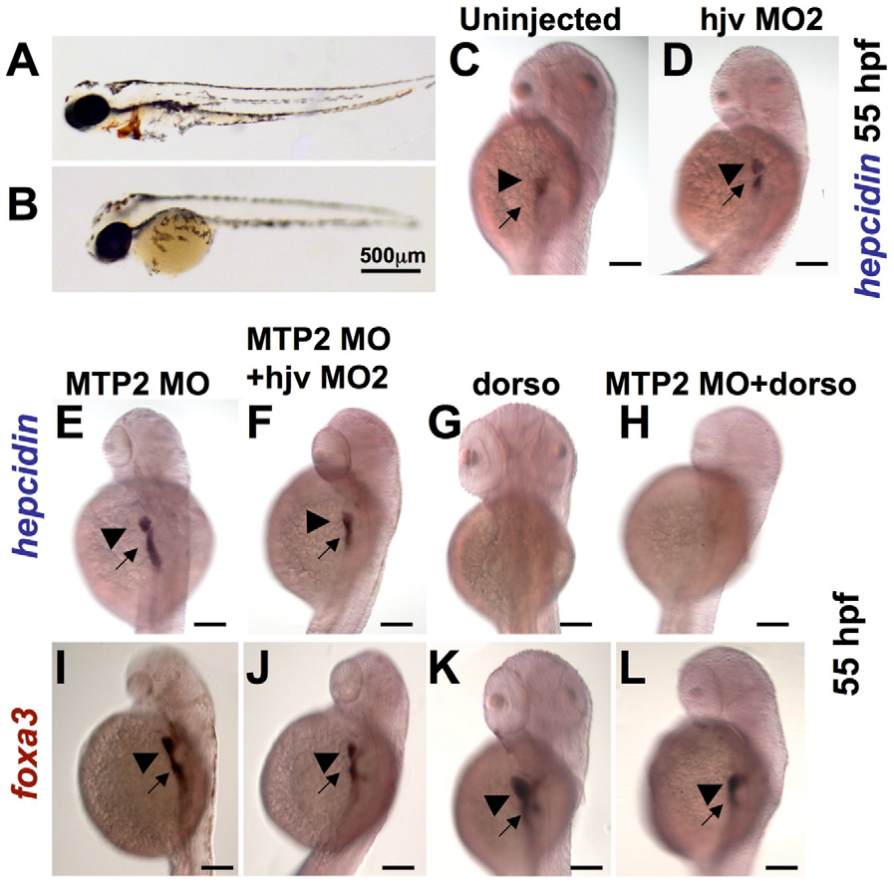
Knockdown of *mtp2* enhances expression of *hepcidin* at 55 hpf. **A–B.** O-dianisidine staining for hemoglobin at 48 hpf demonstrated normal levels of hemoglobin in the cardiac circulation of WT embryos (**A**), but decreased hemoglobin in the *mtp2* morphants (**B**). **C–H.** Whole mount *in situ* hybridization for *hepcidin* at 55 hpf demonstrated normal staining in uninjected controls (**C**) and *hjv* morphants (**D**). Knockdown of *mtp2* (**E**) caused developmental delay, but increased the intensity of *hepcidin* staining in the liver (arrowhead) and the extent and intensity of staining in the intestine (arrow). Co-injection of hjv MO and mtp2 MO (**F**) resulted in a smaller embryo, but preserved *hepcidin* staining in the liver and intestine. Treatment with dorsomorphin from 28–55 hpf abrogated *hepcidin* expression in both uninjected embryos (**G**) and *mtp2* morphants (**H**). **I–L.** Whole mount *in situ* hybridization for *foxa3* demonstrated smaller liver size (arrowhead) in embryos injected with mtp2 MO (**I**,**J**,**L**), compared to dorsomorphin alone (**K**) or untreated embryos (compare with **Figure 3D**). N = 20–30 embryos per group.

As zebrafish *hjv* functioned as a BMP co-receptor in vitro and the message appeared to be present at a low level in embryonic hepatocytes, we hypothesized that *matriptase-2* (*mtp2*) may be inhibiting the effect of *hjv*. Morpholino knockdown of *mtp2* has previously been shown to induce anemia in zebrafish embryos,[17] although *mtp2*′s effect on *hepcidin* expression and the genetic interaction between *mtp2* and *hjv* in zebrafish embryos have not been evaluated previously. Compared to uninjected embryos (Figure 6A), we found that *mtp2* morphants exhibited decreased hemoglobin staining (Figure 6B) at 72 hpf. We also observed a delay in development in the *mtp2* morphants, characterized by a large yolk, decreased embryo size, and decreased melanocyte pigmentation (Figure 6B).

To evaluate the potential interaction of *mtp2* with the BMP pathway and *hjv*, embryos were injected at the one cell stage with mtp2 MO, hjv MO2, or co-injected with hjv MO2 and mtp2 MO, and fixed at 55 hpf for whole mount *in situ* hybridization with probes for *hepcidin* or *foxa3*. Compared to uninjected embryos (Figure 6C) or *hjv* morphants (Figure 6D), *mtp2* morphants exhibited increased staining intensity for *hepcidin* in the foregut, but a smaller area of staining in the liver (Figure 6E). Co-injection of hjv MO1 and mtp2 MO exacerbated the growth retardation, but the embryos exhibited similar *hepcidin* expression in the liver and foregut (Figure 6F) to mtp2 MO alone. Dorsomorphin treatment from 28–55 hpf abrogated *hepcidin* expression in both uninjected embryos (Figure 6G) and in *mtp2* morphants (Figure 6H) indicating that *mtp2* knockdown stimulates *hepcidin* expression in a BMP-dependent manner. Staining with *foxa3* revealed decreased liver size in all the embryos injected with mtp2 MO (Figure 6I, J, and L), compared to dorsomorphin treatment alone (Figure 6K) or no treatment (Figure 5C).

### Knockdown of *mtp2* increases *hepcidin* expression relative to liver size

**Figure 7:**
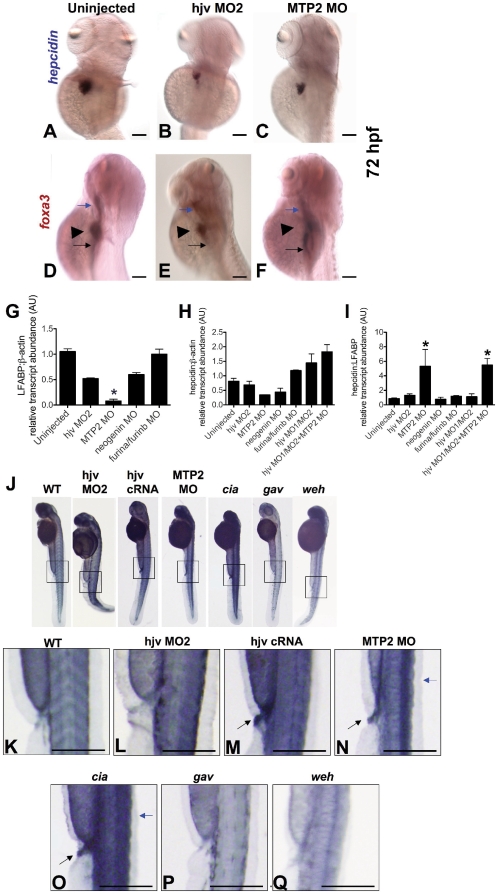
Knockdown of *mtp2* increases *hepcidin* expression and iron staining in zebrafish embryos. **A–F.** Whole mount *in situ* hybridization at 72 hpf for *hepcidin* (dorsolateral) (**A–C**) and *foxa3* (lateral) (**D–F**) in uninjected controls (**A**, **D**), compared to morphants of *hjv* (hjv MO2) (**B**,**E**) or *mtp2* (**C**,**F**). *Foxa3* marking the pharynx (blue arrow), liver (arrowhead), and intestine (black arrow) revealed a smaller liver size in *hjv* morphants (**E**) and particularly in *mtp2* morphants (**F**). N = 40–45 embryos per group. **G–I.** Quantitative real-time RT-PCR for the liver specific marker, *LFABP*, relative to *β-actin* (**G**), and for *hepcidin* relative to the ubiquitous transcript, *β-actin* (**H**) or relative to *LFABP* (**I**). N = 2–8 pools of embryos per group. Data shown are means ± SE. * denotes p<0.05, compared to uninjected controls. **J–Q.** Whole mount nonheme iron staining of zebrafish embryos at 55 hpf with 5x additional magnification of boxed regions. We observed normal iron staining in uninjected WT (**K**) and *hjv* morphants (**L**), but increased iron staining (black arrows) in the somites and proctodeum (terminal gut) of *hjv* cRNA injected (**M**), mtp2 MO injected (**N**), erythroid transferrin receptor deficient mutant *chianti* (*cia*) (**O**), and in the dorsal spinal cord (blue arrows) of *mtp2* morphants and *chianti*. As expected, decreased intraembryonic iron staining was observed in the *transferrin-a* deficient mutant *gavi* (*gav*) (**P**) and in the *ferroportin* deficient mutant weissherbst (*weh*) (**Q**). N = 11–20 embryos per group.

As knockdown of *hjv* and *mtp2* altered embryonic development, we evaluated the effects at 72 hpf to verify if they were similar to those observed at 55 hpf. In comparison to uninjected embryos (Figure 7A), *hjv* morphants (Figure 7B) and *mtp2* morphants (Figure 7C) exhibited smaller areas of *hepcidin* staining at 72 hpf, which correlated with decreased liver size in the morphants, particularly of *mtp2* (Figure 7D–F). The decrease in liver size was supported by quantitative real-time RT-PCR for the liver specific marker, *LFABP* (Figure 7G), which revealed that *LFABP* levels were <10% of normal in *mtp2* morphants (0.08+0.036 vs 1.02+0.069, p = 0.025). In contrast, knockdown of *furina* and *furinb* failed to reduce *LFABP* expression, while knockdown of *hjv* or *neogenin* produced approximately 50% reduction. Quantitative real-time RT-PCR at 72 hpf to assess *hepcidin* transcript levels relative to *β-actin* revealed a decrease in *hepcidin* expression in the *mtp2* morphants (Figure 7H), consistent with the small size of the liver. Normalizing to the liver specific gene, *LFABP*, however, revealed that *mtp2* morphants exhibited a significant increase in *hepcidin* transcript levels compared to uninjected (Figure 7I) (5.31±2.3 vs 0.96±0.04, p<0.05), consistent with increased transcript levels of *hepcidin* in a smaller number of hepatocytes. In contrast, transcript levels of *hepcidin*, normalized to *LFABP*, for morphants of *hjv*, *neogenin*, and *furina/furinb* were not significantly different from uninjected controls. Co-injection of morpholinos for *hjv* and *mtp2* failed to reduce *hepcidin* transcript levels relative to *LFABP* (Figure 7I), indicating that *mtp2*′s effect on *hepcidin* expression does not require *hjv*.

### *Hjv* knockdown fails to increase embryonic nonheme iron stores

*Ferroportin* is localized to the yolk syncytial layer in the zebrafish embryos, where it facilitates the transfer of iron from the yolk into the embryo.[28] We expected that if *hjv* has a significant effect on zebrafish embryonic iron homeostasis, *hjv* morphants would exhibit decreased hepcidin protein levels, which would result in increased ferroportin activity at the yolk syncytial layer and increased embryonic iron stores. Conversely, we expected *hjv* overexpressing embryos or *mtp2* morphants to exhibit decreased embryonic iron stores, secondary to elevated hepcidin levels. We performed staining for nonheme iron at 55 hpf to evaluate these hypotheses (Figure 7J–Q) and found that knock down of *hjv* resulted in a normal level of iron staining (Figure 7J,L), while overexpressing *hjv* increased iron staining in the terminal gut (proctodeum) and somites (Figure 7M), rather than decreasing iron staining. Interestingly, we observed increased iron staining in the somites, proctodeum, brain, and dorsal spinal cord of the *mtp2* morphants (Figure 7J,N) in a pattern of iron accumulation resembling that seen in the erythroid transferrin receptor mutant *chianti* (Figure 7J,O), which has a defect in erythroid iron assimilation. The iron accumulation in the *mtp2* morphants differed from the decreased embryonic iron staining observed in *transferrin-a* deficient *gavi* (Figure 7J,P) and *ferroportin* deficient *weissherbst* (Figure 7J,Q) mutants. Furthermore, treatment with dorsomorphin to suppress *hepcidin* expression failed to rescue the anemia or to reverse the intraembryonic iron accumulation observed in the *mtp2* morphants (Figure S11A–H). Whole mount in situ hybridization for *gata1*, as a marker of erythroid progenitor cells[29], revealed decreased numbers of erythroid progenitor cells in *mtp2* morphants compared to uninjected embryos (Figure S11I,J). These data support the hypothesis that *mtp2* knock down causes anemia in the embryo by decreasing the number of erythroid progenitor cells. Taken together, these data do not support the hypothesis that *hjv* modulates intraembryonic iron stores in zebrafish embryos via effects on *hepcidin*.

## Discussion

We have performed the first detailed analysis of embryonic regulation of *hepcidin* and the role of *hjv* during embryonic development. Previously we demonstrated that *hepcidin* transcript levels in zebrafish embryos increase in response to iron loading[6] and that onset of *hepcidin* expression requires the function of *transferrin-a* and *transferrin receptor 2* [18]. In this study, we found that, as in mammalian models[9], [15], [20], *hepcidin* regulation was responsive to BMP signaling, however, *hjv* (a BMP co-receptor), and the putative *hjv* interacting genes, *furin* and *neogenin*, were not required for *hepcidin* expression in zebrafish embryos. We discovered that knockdown of *matriptase-2* (*mtp2*), a protease which cleaves membrane-bound *hjv* [17], produced anemia, accumulation of intraembryonic iron, and increased *hepcidin* expression in zebrafish embryos, however, surprisingly, *mtp2*′s effect on *hepcidin* expression was independent of *hjv*. Thus the zebrafish embryonic model of *hepcidin* regulation (Figure S12) differs from the mammalian model, which was derived from in vitro studies, human patients, and post-natal animal models. Further studies will be needed to determine if *hepcidin* regulation in mammalian embryos resembles that observed in zebrafish embryos.

### BMP signaling is required for hepcidin expression in zebrafish embryos

Using a heat shock inducible transgenic zebrafish, we found that induction of *bmp2b* increased *hepcidin* expression and phosphorylation of smad1,5, and 8. Dorsomorphin, which specifically inhibits BMP type I receptors, has been previously shown to decrease iron-induced levels of *hepcidin* transcripts and phospho-Smad1,5,8 in adult zebrafish liver, without altering total Smad1 levels.[21] While human BMP4 and BMP9 have been shown to be more potent than BMP2 in stimulating hepcidin transcription in mammalian cell culture[20], recent studies in mouse models[30]–[33] indicate that BMP6, is the most likely physiologic regulator of *hepcidin* transcription in response to iron loading. Among the thirteen BMP genes currently identified in the zebrafish, only BMP2b[34], BMP4[35], and BMP6[36] have been demonstrated to exhibit embryonic endodermal expression. Further studies will be required to determine which BMP is the most critical for the regulation of *hepcidin* expression during zebrafish embryonic development.

### *Hjv* knock down impairs notochord and somite development in zebrafish embryos

In this study we report the first evidence that *hjv* plays a role in notochord and somite development. We found that *hjv* displayed early expression in the notochord and developing somites of zebrafish embryos and knockdown of hjv distorted both structures. The notochord provides structural support to the developing vertebrate embryo and influences somite formation.[37] The flattened, U-shaped somites, observed in *hjv* morphants resembled those seen following knockdown of *neogenin* ([25] and Figure S4), a protein which has been implicated in zebrafish cell migration events and somitogenesis[25] and a binding partner of membrane-bound Hjv[8]. This suggests that Hjv and Neogenin might cooperate to regulate morphogenetic processes within the lateral and paraxial mesoderm, which could explain the defect in liver development observed when *hjv* is knocked down or overexpressed (Figures 5D and 7E).

Although *hjv* is most prominently expressed in the developing somites and skeletal muscle of the mouse embryo[38], [39], *hjv* knock out mouse models have not been reported to exhibit a somite or muscle defect[40], [41]. It is possible that other RGM family members may play a compensatory role for *hjv*. *RGMa* and *RGMb*, although primarily expressed in the central nervous system during mouse embryonic development[38], [39], are detectable in skeletal muscle after birth.[39] The *RGMa* knockout mouse exhibits a partially penetrant failure in cephalic neural tube closure,[39] while an *RGMb* deficient mouse has not been reported.

### *Hjv* is not required for *hepcidin* expression in zebrafish embryos

As we have demonstrated a conserved role for BMP signaling in regulating *hepcidin* expression, we were surprised to find that morpholino knockdown of *hjv* failed to reduce *hepcidin* expression or to increase intraembryonic iron stores. Further supporting an *hjv*-independent regulation of zebrafish embryonic *hepcidin* expression, knock down of *neogenin* or the zebrafish paralogs of *hjv*, failed to decrease *hepcidin* expression relative to liver size. In contrast, the postnatal *hjv* knockout mouse exhibits severe iron overload and low *hepcidin* expression in the liver.[40], [41] The effect of *hjv* deficiency on embryonic *hepcidin* expression and function has not been evaluated in mammalian models.

The lack of an effect on *hepcidin* expression in zebrafish embryos cannot be entirely caused by low levels of *hjv* expression, because overexpression of *hjv* failed to increase *hepcidin* expression. In contrast, overexpression of *bmp2b* readily increased *hepcidin* expression. We cannot exclude a role for *hjv* in regulating *hepcidin* expression in adult zebrafish, particularly as we have demonstrated that zebrafish *hjv* functions as a BMP co-receptor, can activate the human *hepcidin* promoter in vitro, and is expressed, together with *hepcidin* in the zebrafish adult liver. We do not have a model for *hjv* deficiency in adult zebrafish to test this hypothesis. The effect of a morpholino injection dissipates after 4 days of development.

### Mtp2 knockdown increases hepcidin expression independent of *hjv*

We found that the zebrafish *mtp2* morphant embryo exhibits increased *hepcidin* transcript levels relative to the size of its liver and that this effect on *hepcidin* expression is not impaired by knockdown of *hjv*. This contrasts with mouse models in which crossing mice deficient in *matriptase-2* with mice deficient in *hjv* suppresses elevated *hepcidin* (*HAMP*) transcript levels and the microcytic anemia associated with *matriptase-2* deficiency in mice 9–15 weeks of age.[42]

Anemia in *mtp2* morphant zebrafish embryos has been attributed to the effect of excessive *hepcidin* production[17], however we found that abrogation of *hepcidin* expression by treatment with dorsomorphin failed to reverse anemia in *mtp2* morphants (Figure S11A–H). Furthermore, *mtp2* morphants exhibited decreased *gata1* staining, consistent with a decrease in the number of erythroid progenitor cells (Figure S11I,J). The mtp2 morphants also displayed increased intraembryonic iron staining, particularly in the somites, brain, and spinal cord, consistent with the erythroid *transferrin receptor* deficient phenotype (Figure 7O), which is characterized by normal iron transport from the yolk to the embryo, but ineffective transport to the erythrocyte.[43] Thus it seems likely that *mtp2* knockdown produces anemia in zebrafish embryos by decreasing erythroid progenitor development. This, in turn, impairs erythroid iron assimilation, which results in intraembryonic iron loading and an increase in *hepcidin* transcript levels.

The regulation of *hepcidin* has clinical importance for patients with hemochromatosis and thalassemia, who exhibit inappropriately low levels of hepcidin despite the presence of iron overload[44]–[46]. Improving our understanding of *hepcidin* regulation holds promise for better therapies for these patients. The zebrafish embryo has proved a useful tool for identifying and characterizing the function of genes involved in iron metabolism[28], [47]–[49] and elucidating the role of *transferrin* and *transferrin receptor 2* in regulating *hepcidin* expression and development.[18], [43] As *hjv* does not appear to play a role in *hepcidin* regulation in zebrafish embryos, the system will be most useful in identifying *hjv*-independent regulators of *hepcidin* transcription. Future studies will be needed to determine if *hjv* regulates *hepcidin* expression during mammalian development.

## Materials and Methods

### Ethics statement

Ethical approval was obtained from the Institutional Animal Care and Use Committee of Beth Israel Deaconess Medical Center (Animal Welfare Assurance #A3153-01) in accordance with national and international guidelines. Beth Israel Deaconess Medical Center maintains full accreditation from the Association for Assessment and Accreditation of Laboratory Animal Care. *Zebrafish strains*, *maintenance and determination of genotype.* Zebrafish were maintained as described.[50] Tg(*hsp70:bmp2b*) and tg(*hsp70:noggin3*) zebrafish are described elsewhere[19]. Heterozygote carriers of tg(*hsp70:bmp2b*) or tg(*hsp70:noggin3*) were identified by crossing with WT zebrafish, subjecting the progeny embryos at the shield-stage to heat shock at 37°C for 40 min, and assessing the percentage of ventralized or dorsalized embryos produced.[19] Hypochromic anemia mutants used included *chianti* (*cia* ^Tu25f^), *gavi* (*gav* ^IT029^), and *weissherbst* (*weh* ^Tp85c^) [6], [18], [28], [43].

### Bioinformatics

Alignments were generated using ClustalW and Muscle[51], [52], followed by manual refinement using SeaView[53] to remove redundant and improperly annotated sequences. For additional details, please see Figure S6.

### Morpholino Injection, cRNA injection, and Heat Shock

Antisense morpholino oligonucleotides[54], obtained from Gene Tools, Inc. (Philomath, OR), were designed either to interfere with translation or to impair appropriate splicing of transcripts. Morpholinos for *hjv*, *RGMa*, *RGMb*, *RGMd*, *neogenin* [*25*], *furina* [*26*], *furinb* [*26*], and *matriptase-2* [17] (Table S1) were injected at the one-cell stage with 3 nL in 1x Danieau medium, supplemented with phenol red. The aberrant splice produced by injection of hjv MO2 was cloned by PCR amplification with the primers (5′-TCAGTGGTCCGAGCTTCAG-3′ and 5′-CCAACCTGCCGCACTATTAT-3′), cloned into the plasmid pCR2-TOPO (Invitrogen, Carlsbad, CA), and sequenced. The predicted translation was analyzed to identify the signal peptide with the algorithm PrediSi [24]. Full-length zebrafish *hjv* was cloned into the pCS2+ vector. The vector was digested with NotI and sense *hjv* cRNA was synthesized using the SP6 mMachine Kit (Ambion, Austin, TX). The *hjv* cRNA was injected at a concentration of 1000 ng/microliter, similar to the amount of *transferrin receptor 1a* cRNA, which was adequate to rescue transferrin deficiency[18]. Injecting higher concentrations of hjv cRNA was toxic to the embryos. cDNA injections were performed at 50 ng/microliter. For assessment of *bmp2b* expression, embryos at 48 hpf were incubated at 37°C (heat shock) for 40 min and then returned to 28.5°C for 6 hours' incubation or for the duration specified in the time course. The embryos were then transferred to RNAlater (Ambion) or fixed in 4% paraformaldehyde.

### Chemical treatment

Embryos were treated either with 40 µM dorsomorphin [21] dissolved in DMSO or with DMSO only, from 28–55 hpf.

### Whole mount immunohistochemistry

Embryos were fixed overnight at 4°C in 4% paraformaldehyde/1x PBS/0.1% Tween and the staining procedure was performed as described in [55] using Anti-phospho-Smad1/5/8 Antibody (#1511, Cell Signaling Technologies, Danvers, MA) at a dilution of 1∶200 overnight at 4°C. Detection of the primary antibody was performed using biotinylated anti-Rabbit IgG/streptavidin horseradish peroxidase (Rabbit IgG Vectastain Elite Kit #PK-6101,Vector Laboratories, Inc., Burlingame, CA) according to the manufacturer's instructions. Photomicrographs of representative embryos were obtained using an SZX51 zoom stereomicroscope (Olympus, Center Valley, PA) at 40x magnification with a DP-71 camera (Olympus).

### Whole mount *in situ* hybridization

Whole mount *in situ* hybridizations were performed as previously described.[56] The development of endogenous pigments was inhibited by supplementing the embryo medium with 1-phenyl-2-thiourea (PTU) at a final concentration of 0.2 mM. The following antisense riboprobes were generated for use in the *in situ* hybridizations: *hemojuvelin*, *hepcidin* [18], *transferrin-a* [18], *foxa3* [19], *RGMa* [27], *RGMb* [27], *RGMd*, *no tail* (gift of G. Begemann), *myoD* (gift of V. Laudet) and *gata1* [29]. Representative embryos were photographed at 100x magnification with a BX51 compound microscope (Olympus) and a Q-capture 5 digital camera (QImaging, Surrey, BC, Canada). Images were processed using Adobe Photoshop software. Scale bars represent 100 microns, unless otherwise indicated.

### Whole mount embryo staining for cartilage, hemoglobin, and iron

Staining for cartilage was performed with Alcian blue at 5 days post-fertilization following fixation in 4% paraformaldehyde-PBS, as described.[57] Live anesthetized embryos were stained for hemoglobin with *o*-dianisidine, as described.[58] Diaminobenzidine (DAB) enhanced-staining for ferric iron was performed as described[59] following fixation in 4% paraformaldehyde-PBS. Photomicrographs of representative embryos were obtained using an SZX51 zoom stereomicroscope (Olympus) at 40x magnification with a DP-71 camera (Olympus).

### Quantitative analysis of gene expression

At specified time points, embryos were pooled in groups of 20, anesthetized with tricaine, and placed in RNAlater (Ambion). RNA extraction, generation of cDNA, and quantitative real-time RT-PCR assay were performed as previously described.[6], [60] Detection and analysis were performed on an ABI 7000 and an ABI 7700 (Applied Biosystems, Inc.). Data presented are the means and standard errors. N = 2–8 pools per time point or condition. For additional details, please see supplemental Methods S1.

### Flow cytometry

Transgenic embryos expressing green fluorescent protein (GFP) under the control of the zebrafish *liver fatty acid binding protein* (*LFABP*) promoter (tg(*LFABP*:*GFP*)) were a gift from W. Goessling. The embryos were manually dissociated in 0.9% PBS and sorted for fluorescence using a 488 nm laser with a FACSAria II (BD Biosciences, San Jose, CA). N = 80–100 embryos for each sorting.

### Biostatistical Analysis

Heterogeneity among cohorts was analyzed by ANOVA using Prism 5 (GraphPad Software, Inc., San Diego, CA). Tests for heterogeneity used the natural log for assessment of transcript levels. All estimates and standard errors presented have been converted back to the original units. When the global P-value obtained from the ANOVA analysis was statistically significant, pairwise comparisons between the cohorts were performed using two-tailed Student's t-tests with a Bonferroni correction for multiple comparisons. P values less than 0.05 were deemed statistically significant and are indicated by an asterisk.

### Luciferase Assays

Human hepatoma (Hep3B) cells were cultured in Dulbecco's Modification of Eagle's Medium (Cellgro, Mediatech Inc., Virginia) supplemented with 10% Fetal Bovine Serum at 37°C in 5% CO_2_. All transfections were performed with Lipofectamine-2000 (Invitrogen Life Technologies, Carlsbad, CA). Hep3B cells were transiently transfected with zebrafish *hjv* cRNA (0–5000 ng) and pGL2-2.7 Hepc, a 2.7 kb fragment of the human hepcidin promoter upstream of the Firefly luciferase reporter gene, or a plasmid containing the BMP response element (BRE) upstream of a Firefly luciferase reporter gene.[9] A control pRL-TK Renilla luciferase reporter (Promega, Madison, NY) was also transiently transfected simultaneously, to control for transfection efficiency. The cells were incubated in the presence or absence of BMP6 (5 ng/ml) (R&D Systems, Minneapolis, MN) for sixteen hours and then lysed. The luciferase activity was determined with the Dual Reporter Assay (Promega, Madison, NY).

## Supporting Information

Table S1 (0.07 MB PDF)

Methods S1 (0.20 MB PDF)

Figure S1 Treatment with dorsomorphin decreases BMP2b-induced phospho-smad1,5,8 staining in zebrafish embryos. Tg(hsp70:bmp2b) embryos were fixed at 55 hpf for immunohistochemical staining for phospho-smad1,5,8 following (A) no heat shock and no chemical treatment (−HS, −dorso), (B) no heat shock, but treatment with dorsomorphin (−HS, +dorso), (C) heat shock and no chemical treatment (+HS, −dorso), (D) heat shock and treatment with dorsomorphin (+HS, +dorso), representative embryos lateral view. Heat shock was performed at 48 hpf. Dorsomorphin treatment was performed from 28–55 hpf at a concentration of 40 µM. For enhanced sensitivity, a fluorescently-labeled secondary antibody was used (Alexa Fluor® 488 goat anti-rabbit IgG, Invitrogen, #A-11008). Embryos were illuminated with an X-cite Series 120 PC microscope lamp (Exfo Life Sciences and Industrial Division, Quebec, Canada) and emitted light was filtered with a green fluorescent protein (GFP) filter set. N = 15–22 embryos per group.
(2.47 MB TIF)

Figure S2 Knock down of hjv fails to produce anemia in zebrafish embryos. O-dianisidine staining for hemoglobin in embryos at 50 hpf, which were either uninjected (A) or injected with hjv MO2 (B) (lateral view). N = 42 embryos per group.
(0.87 MB TIF)

Figure S3 Knock down of hjv interacting proteins, neogenin or furin, fails to decrease hepcidin expression. Whole mount in situ hybridization for hepcidin (A–C, blue arrow) and foxa3 (D–F, black arrowhead) in uninjected embryos (A,D), compared to embryos injected with neogenin MO (B,E) or morpholinos directed against both zebrafish furins (furina and furinb) (C,F), dorsolateral view. N = 20 embryos per group.
(0.98 MB TIF)

Figure S4 Neogenin knockdown reproduced the reported defect in somitogenesis associated with neogenin deficiency. A,B. Whole mount in situ hybridization for myoD to stain the somites in uninjected (A) and neogenin morphants (B) at the 20 somites' stage of development (dorsal view) confirmed that injection of the neogenin morpholino at 0.15 mM produced elongation of the somites, manifest by increased distance between the two arrowheads. This is characteristic of the neogenin deficient phenotype, as described by [4]. Scale bar represents 100 microns. C,D. Whole mount in situ hybridization for hepcidin at 72 hpf in uninjected control embryos (C) and neogenin morphants (D) (lateral view) revealed a shortened body axis with a curved tail and flattened somites (arrowhead) in the neogenin morphants. Hepcidin expression is present in the liver (arrow) of the neogenin morphant, although the expression domain of hepcidin is smaller than in the uninjected control. Scale bar represents 200 microns. N = 20 embryos per group. Embryos were photographed at 100x magnification with a an Axio Imager 1 compound microscope (Carl Zeiss MicroImaging, Inc., Thornwood, NY) and an AxioCam ICc1 digital camera (Carl Zeiss MicroImaging, Inc.) (A,B) or a BX51 compound microscope (Olympus, Center Valley, PA) and a Q-capture 5 digital camera (QImaging, Surrey, BC, Canada) (C,D).
(3.73 MB TIF)

Figure S5 Whole mount Alcian blue staining for cartilage in zebrafish embryos at 5 days post-fertilization confirms a branchial arch phenotype in furin morphants. Dorsolateral view of the head of an uninjected control embryo (A) and an embryo injected with morpholinos to knock down furina and furinb (B) reveals an open mouth phenotype (arrow in B) in the furina/furinb morphant. Lateral view of an uninjected control (C) and a furina/furinb morphant showing the fused cartilage elements (arrowhead in D) characteristic of furin morphants. N = 20 embryos per group.
(3.46 MB TIF)

Figure S6 Phylogeny and expression of zebrafish RGM's. Phylogenetic tree (A) of hjv and repulsive guidance molecule genes (RGM's) in chordates. The four zebrafish RGM paralogs are highlighted in red. Hjv is also known as RGMc. B–I. Whole mount in situ hybridization of zebrafish embryos, dorsolateral views, at 50 hpf (B,D,F,H) and 72 hpf (C,E,G,I), for RGMa (B,C), RGMb (D,E), hjv (F,G), and RGMd (H,I) revealed that none of the RGM genes are detectable in the developing liver. Strong staining was detected in the mid and hindbrain for RGMa at 50 hpf (B) and 72 hpf (C, black arrows). At 50 hpf (D) and 72 hpf (E), RGMb is faintly expressed in the mid and hindbrain (black arrows). At 50 and 72 hpf, hemojuvelin is no longer detected in the developing embryo by in situ hybridization (F,G). At 50 hpf, RGMd transcripts were detected in the pharyngeal arches (H, black arrow). RGMd expression was no longer detected at 72 hpf (I). N = 20 embryos per group. (J) Phylogenetic tree of the RGM gene family constructed with all available vertebrate sequences. Note that hjv is expressed in a wide range of mammals, fish, and in Xenopus. We have identified hjv in the genome of a bird, the zebra finch (arrow), for the first time. RGMd has only been identified in fish. To generate the tree shown, we downloaded the protein sequences of the RGM gene families defined in the Ensembl database version 52 (as of December 2008) (<http://www.ensembl.org/>), which includes the hjv sequences. In addition to the Ensembl data, which also includes the Uniprot database (<http://www.uniprot.org/>), we also screened the NCBI database (<http://www.ncbi.nlm.nih.gov/>). Alignments were generated using ClustalW and Muscle[5], [6], followed by manual refinement using SeaView[7] to remove redundant and improperly annotated sequences. Phylogenetic tree reconstruction was carried out using the maximum likelihood (ML) method. Of note, the neighbor-joining (NJ) method[7] gives the same basal node topology. For ML analyses, robustness of the obtained tree topologies was assessed with 1000 bootstrap replicates; those below 50% are not shown. The NJ tree was constructed with Phylo_Win using a Poisson correction and pairwise gap removal[7]. The ML tree was obtained with PhyML[8] using a JTT model[9], a discrete gamma model with 4 categories. The gamma shape parameter was estimated by ML and the proportion of invariable sites was also estimated by ML.
(2.35 MB TIF)

Figure S7 Effect of morpholino knockdown of RGM genes at 55 hpf. Whole mount in situ hybridization for hepcidin (A–D) or foxa3 (E–H), dorsolateral views. Compared to uninjected controls (A), knockdown of RGMa (B), RGMb (C), or RGMd (D) failed to inhibit hepcidin expression (arrow). E–H. Expression of foxa3 in the liver (arrowhead) revealed a slight reduction of liver size in the morphants (F–H) compared to control (E). N = 20 embryos per group.
(2.64 MB TIF)

Figure S8 Effect of knockdown of RGM genes at 72 hpf. Whole mount in situ hybridization for hepcidin (A–D) or foxa3 (E–H), dorsolateral views. Compared to uninjected controls (A), knockdown of RGMa (B), RGMb (C), or RGMd (D) failed to inhibit hepcidin expression. E–H. Expression of foxa3 in the liver revealed a significant reduction of liver size in the RGMb and RGMd morphants (G, H). N = 20 embryos per group. I. Quantitative real-time RT-PCR revealed no significant decrease in hepcidin transcript levels relative to liver fatty acid binding protein (LFABP). N = 3 pools of embryos per group. Data shown are means + SE.
(2.43 MB TIF)

Figure S9 Additional expression data for zebrafish embryonic hepatocytes and zebrafish adult tissues. A. Quantitative real-time RT-PCR to assess transcript levels of LFABP (liver fatty acid binding protein) relative to β-actin in hepatocytes sorted from pools of 80–100 transgenic zebrafish embryos at 72 hpf. N = 2 pools per group. Data shown are means +/− SE. * indicates p<0.05 compared to unsorted. B. Semiquantitative RT-PCR for hepcidin, RGMa, RGMb, hjv, RGMd, and neogenin performed with RNA from adult zebrafish liver and skeletal muscle. Hepcidin expression was detected in the adult liver, but not in adult skeletal muscle. All RGM genes and neogenin were detected in the adult liver and skeletal muscle.
(1.21 MB TIF)

Figure S10 Effect of injecting zebrafish hjv cDNA in zebrafish embryos. pHjv-CS2 or pCS2 vector only (50 ng/microliter) were each injected into zebrafish embryos at the one cell stage. Quantitative real-time RT-PCR for hepcidin transcript levels normalized to β-actin expression revealed no significant increase in hepcidin expression at 55 hpf in embryos injected with pHjv-CS2 cDNA compared to pCS2 vector alone. N = 5–6 pools per group. Data shown are means +/− SE.
(0.72 MB TIF)

Figure S11 Effect of dorsomorphin on anemia and iron loading in mtp2 deficient embryos. Embryos were injected with mtp2 morpholino at the one cell stage, followed by treatment with dorsomorphin from 28 hpf until fixation for either o-dianisidine staining at 50 hpf (A–D) or whole mount nonheme iron staining at 55 hpf (E–H), lateral views. Uninjected controls (A) and embryos treated with dorsomorphin (B) exhibited normal hemoglobin staining, while mtp2 morphants (C) manifest decreased hemoglobin staining, which failed to improve when mtp2 morphants were treated with dorsomorphin (D). N = 54–99 embryos per group. Compared to uninjected controls (E), embryos treated with dorsomorphin (F), mtp2 morphants (G), or mtp2 morphants treated with dorsomorphin (H) exhibited increased iron staining in the somites, brain, and dorsal spinal cord. N = 32–45 embryos per group. (I,J) Whole mount in situ hybridization for gata1 (lateral views) when embryos have developed 24 somites, about 22 hpf, demonstrated decreased numbers of gata1-staining erythroid precursors in mtp2 morphants compared to uninjected embryos. N = 21–36 embryos per group.
(3.25 MB TIF)

Figure S12 Comparison of the role of hemojuvelin in the mammalian model of hepcidin regulation with the zebrafish embryonic model. A. In the mammalian model of hepcidin regulation, which is based on in vitro studies, human patients, and post-natal animal studies[10]–[25], hjv acts as a BMP co-receptor to promote BMP signaling, which results in increased hepcidin transcription. Cleavage of membrane-bound hjv by matriptase-2 or furin results in the release of soluble hjv, which acts as a competitive inhibitor for BMP signaling. B. In the zebrafish embryonic model, which we have developed, BMP signaling promotes hepcidin transcription independent of hjv. Matriptase-2 exhibits a BMP-dependent, but hjv-independent effect on hepcidin expression. Stimulatory effects are shown by arrows. Repressive effect is shown by -|.
(1.03 MB TIF)
