# Supplementary material for: BMP Signaling Modulates Hepcidin Expression in Zebrafish Embryos Independent of Hemojuvelin
Source: PLoS One. 2011 Jan 21;6(1):e14553. doi: 10.1371/journal.pone.0014553 (PMC3024971; doi:10.1371/journal.pone.0014553)
Supplement: Table S1 — (0.07 MB PDF) [file pone.0014553.s001.pdf]

Table S1. Sequences of morpholinos used to knockdown *hjv* and other genes. Exon is abbreviated by E. Mismatch substitutions are shown in lower-case. Sequences targeting the ATG are underlined. Concentrations injected to achieve the reported phenotypes are indicated.

Morpholino	Target	Sequence	Concentration
hjv MO1	ATG	5'-CTGATGCTGCCATTCCCATCCATAC-3'	0.2 mM
hjv MO2	E2 donor	5'-AAATTGAATTTTTACCTGATGGAGC-3'	0.2 mM
hjv MMO2	mismatch	5'-AAATTcAAaTTTTAgCTcATcGAGC-3'	0.2 mM
furina MO[1]	E9	5'-GAGGGACTCACAATCTGTTTCTCAT-3'	0.2 mM
furinb MO[1]	E9	5'-AGCCCAGATCGACCCTAGAAACACA-3'	0.2 mM
neogenin MO[2]	ATG	5'-GGCTCCCCGCTCCGCCATCACTTTA-3'	0.15 mM
mtp2 MO[3]	E3 acceptor	5'-GAACACCGCCATCTGAAAACAAATA-3'	0.5 mM
RGMa MO	5'-UTR	5'-ACACTTTTGGGTTGAGTTTCTTTTGC-3'	0.2 mM
RGMb MO	ATG	5'-AGATCCTGCTCTCCCCATACCCATC-3'	0.2 mM
RGMd MO	ATG	5'-GTTGAGAGTGCCGCTTCTCCCCATG-3'	0.2 mM

References

1. Walker MB, Miller CT, Coffin Talbot J, Stock DW, Kimmel CB (2006) Zebrafish furin mutants reveal intricacies in regulating Endothelin1 signaling in craniofacial patterning. Dev Biol 295: 194-205.
2. Mawdsley DJ, Cooper HM, Hogan BM, Cody SH, Lieschke GJ, et al. (2004) The Netrin receptor Neogenin is required for neural tube formation and somitogenesis in zebrafish. Dev Biol 269: 302-315.
3. Silvestri L, Pagani A, Nai A, De Domenico I, Kaplan J, et al. (2008) The serine protease matriptase-2 (TMPRSS6) inhibits hepcidin activation by cleaving membrane hemojuvelin. Cell Metab 8: 502-511.
