# Supplementary material for: BMP Signaling Modulates Hepcidin Expression in Zebrafish Embryos Independent of Hemojuvelin
Source: PLoS One. 2011 Jan 21;6(1):e14553. doi: 10.1371/journal.pone.0014553 (PMC3024971; doi:10.1371/journal.pone.0014553)
Supplement: Methods S1 — (0.20 MB PDF) [file pone.0014553.s002.pdf]

Supplemental Methods

Quantitative analysis of gene expression. At specified time points, embryos were pooled in groups of 20, anesthetized with tricaine, and placed in RNAlater (Ambion). RNA extraction, generation of cDNA, and multiplex quantitative real-time RT-PCR assay for *hepcidin* normalized to *β-actin* expression, was performed as previously described.[1] Quantitative real-time RT-PCR for *bmp2b* or *LFABP* expression was performed by amplifying with primers BMP2b-F 5'-CCAGCAGAGCAAACACGATA-3' and BMP2b-R 5'-ACTGCTGCGTTGTTTTTCCT-3' or *LFABP* primers[2] with 1x SYBR Green Master Mix (Applied Biosystems, Inc., Foster City, CA), according to the manufacturer's instructions. For normalization of expression, quantitative PCR for *β-actin* was performed in parallel SYBR green reactions. Detection and analysis were performed on an ABI 7000 and an ABI 7700 (Applied Biosystems, Inc.). Transcript abundance was expressed as a fold-increase over calibrator, according to the method of [3]. The calibrator group is in the first column of each graph. Data presented are the means and standard errors. N=2-8 pools per time point or condition. Primers for *hjv* semiquantitative RT-PCR were hjv-F 5'-TCAGTGGTCCGAGCTTCAG-3' and hjv-R 5'-CCAACCTGCCGCACTATTAT-3'. The cycling parameters were as follows: 94°C for 20 seconds, 60°C for 30 seconds, 72°C for 1 min, for 27 cycles.

Supplementary Figure Legends

Figure S1. Treatment with dorsomorphin decreases BMP2b-induced phospho-smad1,5,8 staining in zebrafish embryos. Tg(*hsp70*:*bmp2b*) embryos were fixed at 55 hpf for immunohistochemical staining for phospho-smad1,5,8 following **(A)** no heat shock and no chemical treatment (-HS, -dorso), **(B)** no heat shock, but treatment with dorsomorphin (-HS, +dorso), **(C)** heat shock and no chemical treatment (+HS, -dorso), **(D)** heat shock and treatment with dorsomorphin (+HS, +dorso), representative embryos lateral view. Heat shock was performed at 48 hpf. Dorsomorphin treatment was performed from 28-55 hpf at a concentration of 40 μM. For enhanced sensitivity, a fluorescently-labeled secondary antibody was used (Alexa Fluor® 488 goat anti-rabbit IgG, Invitrogen, #A-11008). Embryos were illuminated with an X-cite Series 120 PC microscope lamp (Exfo Life Sciences and Industrial Division, Quebec, Canada) and emitted light was filtered with a green fluorescent protein (GFP) filter set. N=15-22 embryos per group.

Figure S2. Knock down of *hjv* fails to produce anemia in zebrafish embryos. O-dianisidine staining for hemoglobin in embryos at 50 hpf, which were either uninjected (**A**) or injected with hjv MO2 (**B**) (lateral view). N=42 embryos per group.

Figure S3. Knock down of *hjv* interacting proteins, neogenin or furin, fails to decrease hepcidin expression. Whole mount in situ hybridization for *hepcidin* (**A-C**) and *foxa3* (**D-F**) in uninjected embryos (**A,D**), compared to embryos injected with neogenin MO (**B,E**) or morpholinos directed against both zebrafish furins (*furina* and

furinb) (**C,F**), dorsolateral view. N=20 embryos per group.

Figure S4. ***Neogenin*** **knockdown reproduced the reported defect in somitogenesis associated with neogenin deficiency. A,B.** Whole mount *in situ* hybridization for *myoD* to stain the somites in uninjected (**A**) and *neogenin* morphants (**B**) at the 20 somites' stage of development (dorsal view) confirmed that injection of the neogenin morpholino at 0.15 mM produced elongation of the somites, manifest by increased distance between the two arrowheads.   This is characteristic of the neogenin deficient phenotype, as described by [4]. Scale bar represents 100 microns. **C,D.** Whole mount in situ hybridization for *hepcidin* at 72 hpf in uninjected control embryos (**C**) and *neogenin* morphants (**D**) (lateral view) revealed a shortened body axis with a curved tail and flattened somites (arrowhead) in the *neogenin* morphants. *Hepcidin* expression is present in the liver (arrow) of the neogenin morphant, although the expression domain of *hepcidin* is smaller than in the uninjected control. Scale bar represents 200 microns. N=20 embryos per group. Embryos were photographed at 100x magnification with a an Axio Imager 1 compound microscope (Carl Zeiss MicroImaging, Inc., Thornwood, NY) and an AxioCam ICc1 digital camera (Carl Zeiss MicroImaging, Inc.) (**A,B**) or a BX51 compound microscope (Olympus, Center Valley, PA) and a Q-capture 5 digital camera (QImaging, Surrey, BC, Canada) (**C,D**).

Figure S5. Whole mount Alcian blue staining for cartilage in zebrafish embryos at 5 days post-fertilization confirms a branchial arch phenotype in ***furin*** **morphants.** Dorsolateral view of the head of an uninjected control embryo (**A**) and an embryo

injected with morpholinos to knock down *furina* and *furinb* (**B**) reveals an open mouth phenotype (arrow in **B**) in the *furina/furinb* morphant. Lateral view of an uninjected control (**C**) and a *furina/furinb* morphant showing the fused cartilage elements (arrowhead in **D**) characteristic of *furin* morphants. N=20 embryos per group.

Figure S6. **Phylogeny and expression of zebrafish RGM's.** Phylogenetic tree (**A**) of *hjv* and repulsive guidance molecule genes (RGM's) in chordates. The four zebrafish RGM paralogs are highlighted in red. *Hjv* is also known as *RGMc*. **B-I.** Whole mount *in situ* hybridization of zebrafish embryos, dorsolateral views, at 50 hpf (**B, D, F, H**) and 72 hpf (**C,E,G,I)**, for *RGMa* (**B,C**), *RGMb* (**D,E**), *hjv* (**F,G**), and *RGMd* (**H,I**) revealed that none of the RGM genes are detectable in the developing liver. Strong staining was detected in the mid and hindbrain for *RGMa* at 50 hpf (**B**) and 72 hpf (**C,** black arrows). At 50 hpf (**D**) and 72 hpf (**E**), *RGMb* is faintly expressed in the mid and hindbrain (black arrows). At 50 and 72 hpf, hemojuvelin is no longer detected in the developing embryo by *in situ* hybridization (**F**,**G**). At 50 hpf, *RGMd* transcripts were detected in the pharyngeal arches (**H**, black arrow). *RGMd* expression was no longer detected at 72 hpf (**I**). N=20 embryos per group. (**J**) Phylogenetic tree of the RGM gene family constructed with all available vertebrate sequences. Note that *hjv* is expressed in a wide range of mammals, fish, and in *Xenopus*. We have identified *hjv* in the genome of a bird, the zebra finch (arrow), for the first time. *RGMd* has only been identified in fish. To generate the tree shown, we downloaded the protein sequences of the RGM gene families defined in the Ensembl database version 52 (as of December 2008) (<http://www.ensembl.org/ >), which includes the *hjv* sequences. In addition to the

Ensembl data, which also includes the Uniprot database (<http://www.uniprot.org/>), we also screened the NCBI database (<http://www.ncbi.nlm.nih.gov/>). Alignments were generated using ClustalW and Muscle[5,6], followed by manual refinement using SeaView[7] to remove redundant and improperly annotated sequences. Phylogenetic tree reconstruction was carried out using the maximum likelihood (ML) method. Of note, the neighbor-joining (NJ) method[7] gives the same basal node topology. For ML analyses, robustness of the obtained tree topologies was assessed with 1000 bootstrap replicates; those below 50% are not shown. The NJ tree was constructed with Phylo_Win using a Poisson correction and pairwise gap removal[7]. The ML tree was obtained with PhyML[8] using a JTT model[9], a discrete gamma model with 4 categories. The gamma shape parameter was estimated by ML and the proportion of invariable sites was also estimated by ML.

Figure S7. Effect of morpholino knockdown of RGM genes at 55 hpf. Whole mount in situ hybridization for *hepcidin* (**A-D**) or *foxa3* (**E-H**), dorsolateral views. Compared to uninjected controls (**A**), knockdown of *RGMa* (**B**), *RGMb* (**C**), or *RGMd* (**D**) failed to inhibit *hepcidin* expression (arrow). **E-H.** Expression of *foxa3* in the liver (arrowhead) revealed a slight reduction of liver size in the morphants (**F-H**) compared to control (**E**). N=20 embryos per group.

Figure S8. **Effect of knockdown of RGM genes at 72 hpf**. Whole mount in situ hybridization for *hepcidin* (**A-D**) or *foxa3* (**E-H**), dorsolateral views. Compared to uninjected controls (**A**), knockdown of *RGMa* (**B**), *RGMb* (**C**), or *RGMd* (**D**) failed to

inhibit *hepcidin* expression. **E-H.** Expression of *foxa3* in the liver revealed a significant reduction of liver size in the *RGMb* and *RGMd* morphants (**G, H**). N=20 embryos per group. **I.** Quantitative real-time RT-PCR revealed no significant decrease in *hepcidin* transcript levels relative to *liver fatty acid binding protein* (*LFABP*). N=3 pools of embryos per group. Data shown are means ± SE.

Figure S9. **Additional expression data for zebrafish embryonic hepatocytes and zebrafish adult tissues. A.** Quantitative real-time RT-PCR to assess transcript levels of *LFABP* (liver fatty acid binding protein) relative to *β-actin* in hepatocytes sorted from pools of 80-100 transgenic zebrafish embryos at 72 hpf. N=2 pools per group. Data shown are means ± SE. * indicates $p<0.05$ compared to unsorted. **B.** Semiquantitative RT-PCR for *hepcidin*, *RGMa*, *RGMb*, *hjv*, *RGMd*, and *neogenin* performed with RNA from adult zebrafish liver and skeletal muscle. *Hepcidin* expression was detected in the adult liver, but not in adult skeletal muscle. All RGM genes and *neogenin* were detected in the adult liver and skeletal muscle.

Figure S10. Effect of injecting zebrafish *hjv* cDNA in zebrafish embryos. pHjv-CS2 or pCS2 vector only (50 ng/microliter) were each injected into zebrafish embryos at the one cell stage. Quantitative real-time RT-PCR for *hepcidin* transcript levels normalized to *β-actin* expression revealed no significant increase in *hepcidin* expression at 55 hpf in embryos injected with pHjv-CS2 cDNA compared to pCS2 vector alone. N=5-6 pools per group. Data shown are means ± SE.

Figure S11. Effect of dorsomorphin on anemia and iron loading in *mtp2* deficient embryos. Embryos were injected with *mtp2* morpholino at the one cell stage, followed by treatment with dorsomorphin from 28 hpf until fixation for either o-dianisidine staining at 50 hpf (**A-D**) or whole mount nonheme iron staining at 55 hpf (**E-H**), lateral views. Uninjected controls (**A**) and embryos treated with dorsomorphin (**B**) exhibited normal hemoglobin staining, while *mtp2* morphants (**C**) manifest decreased hemoglobin staining, which failed to improve when *mtp2* morphants were treated with dorsomorphin (**D**). N=54-99 embryos per group. Compared to uninjected controls (**E**), embryos treated with dorsomorphin (**F**), *mtp2* morphants (**G**), or *mtp2* morphants treated with dorsomorphin (**H**) exhibited increased iron staining in the somites, brain, and dorsal spinal cord. N=32-45 embryos per group. (**I,J**) Whole mount *in situ* hybridization for *gata1* (lateral views) when embryos have developed 24 somites, about 22 hpf, demonstrated decreased numbers of *gata1*-staining erythroid precursors in *mtp2* morphants compared to uninjected embryos. N=21-36 embryos per group.

Figure S12. Comparison of the role of hemojuvelin in the mammalian model of hepcidin regulation with the zebrafish embryonic model. A. In the mammalian model of *hepcidin* regulation, which is based on in vitro studies, human patients, and post-natal animal studies[10-25], *hjv* acts as a BMP co-receptor to promote BMP signaling, which results in increased *hepcidin* transcription. Cleavage of membrane-bound *hjv* by *matriptase-2* or *furin* results in the release of soluble *hjv*, which acts as a competitive inhibitor for BMP signaling. **B.** In the zebrafish embryonic model, which we have developed, BMP signaling promotes *hepcidin* transcription independent of *hjv*.

Matriptase-2 exhibits a BMP-dependent, but *hjv*-independent effect on *hepcidin* expression. Stimulatory effects are shown by arrows. Repressive effect is shown by -|.

Supplementary References

1. Fraenkel PG, Traver D, Donovan A, Zahrieh D, Zon LI (2005) Ferroportin1 is required for normal iron cycling in zebrafish. J Clin Invest 115: 1532-1541.
2. Goessling W, North TE, Lord AM, Ceol C, Lee S, et al. (2008) APC mutant zebrafish uncover a changing temporal requirement for wnt signaling in liver development. Dev Biol 320: 161-174.
3. Trant JM, Gavasso S, Ackers J, Chung BC, Place AR (2001) Developmental expression of cytochrome P450 aromatase genes (CYP19a and CYP19b) in zebrafish fry (Danio rerio). J Exp Zool 290: 475-483.
4. Mawdsley DJ, Cooper HM, Hogan BM, Cody SH, Lieschke GJ, et al. (2004) The Netrin receptor Neogenin is required for neural tube formation and somitogenesis in zebrafish. Dev Biol 269: 302-315.
5. Edgar RC (2004) MUSCLE: a multiple sequence alignment method with reduced time and space complexity. BMC Bioinformatics 5: 113.
6. Larkin MA, Blackshields G, Brown NP, Chenna R, McGettigan PA, et al. (2007) Clustal W and Clustal X version 2.0. Bioinformatics 23: 2947-2948.
7. Galtier N, Gouy M, Gautier C (1996) SEAVIEW and PHYLO_WIN: two graphic tools for sequence alignment and molecular phylogeny. Comput Appl Biosci 12: 543-548.

8. Guindon S, Gascuel O (2003) A simple, fast, and accurate algorithm to estimate large phylogenies by maximum likelihood. Syst Biol 52: 696-704.

9. Jones DT, Taylor WR, Thornton JM (1992) The rapid generation of mutation data matrices from protein sequences. Comput Appl Biosci 8: 275-282.

10. Babitt JL, Huang FW, Wrighting DM, Xia Y, Sidis Y, et al. (2006) Bone morphogenetic protein signaling by hemojuvelin regulates hepcidin expression. Nat Genet 38: 531-539.

11. Babitt JL, Huang FW, Xia Y, Sidis Y, Andrews NC, et al. (2007) Modulation of bone morphogenetic protein signaling in vivo regulates systemic iron balance. J Clin Invest 117: 1933-1939.

12. De Domenico I, Ward DM, Kaplan J (2007) Hepcidin regulation: ironing out the details. J Clin Invest 117: 1755-1758.

13. Huang FW, Pinkus JL, Pinkus GS, Fleming MD, Andrews NC (2005) A mouse model of juvenile hemochromatosis. J Clin Invest 115: 2187-2191.

14. Lin L, Goldberg YP, Ganz T (2005) Competitive regulation of hepcidin mRNA by soluble and cell-associated hemojuvelin. Blood 106: 2884-2889.

15. Lin L, Nemeth E, Goodnough JB, Thapa DR, Gabayan V, et al. (2008) Soluble hemojuvelin is released by proprotein convertase-mediated cleavage at a conserved polybasic RNRR site. Blood Cells Mol Dis 40: 122-131.

16. Niederkofler V, Salie R, Arber S (2005) Hemojuvelin is essential for dietary iron sensing, and its mutation leads to severe iron overload. J Clin Invest 115: 2180-2186.

17. Papanikolaou G, Samuels ME, Ludwig EH, MacDonald ML, Franchini PL, et al. (2004) Mutations in HFE2 cause iron overload in chromosome 1q-linked juvenile hemochromatosis. Nat Genet 36: 77-82.

18. Ramsay AJ, Hooper JD, Folgueras AR, Velasco G, Lopez-Otin C (2009) Matriptase-2 (TMPRSS6): a proteolytic regulator of iron homeostasis. Haematologica 94: 840-849.

19. Silvestri L, Guillem F, Pagani A, Nai A, Oudin C, et al. (2009) Molecular mechanisms of the defective hepcidin inhibition in TMPRSS6 mutations associated with iron-refractory iron deficiency anemia. Blood 113: 5605-5608.

20. Silvestri L, Pagani A, Camaschella C (2008) Furin-mediated release of soluble hemojuvelin: a new link between hypoxia and iron homeostasis. Blood 111: 924-931.

21. Silvestri L, Pagani A, Fazi C, Gerardi G, Levi S, et al. (2007) Defective targeting of hemojuvelin to plasma membrane is a common pathogenetic mechanism in juvenile hemochromatosis. Blood 109: 4503-4510.

22. Silvestri L, Pagani A, Nai A, De Domenico I, Kaplan J, et al. (2008) The serine protease matriptase-2 (TMPRSS6) inhibits hepcidin activation by cleaving membrane hemojuvelin. Cell Metab 8: 502-511.

23. Truksa J, Peng H, Lee P, Beutler E (2006) Bone morphogenetic proteins 2, 4, and 9 stimulate murine hepcidin 1 expression independently of Hfe, transferrin receptor 2 (Tfr2), and IL-6. Proc Natl Acad Sci U S A 103: 10289-10293.

24. Yu PB, Hong CC, Sachidanandan C, Babitt JL, Deng DY, et al. (2008) Dorsomorphin inhibits BMP signals required for embryogenesis and iron metabolism. Nat Chem Biol 4: 33-41.

25. Zhang AS, Anderson SA, Meyers KR, Hernandez C, Eisenstein RS, et al. (2007) Evidence that inhibition of hemojuvelin shedding in response to iron is mediated through neogenin. J Biol Chem 282: 12547-12556.
