# Supplementary figures and images for: BMP Signaling Modulates Hepcidin Expression in Zebrafish Embryos Independent of Hemojuvelin
Source: PLoS One. 2011 Jan 21;6(1):e14553. doi: 10.1371/journal.pone.0014553 (PMC3024971; doi:10.1371/journal.pone.0014553)

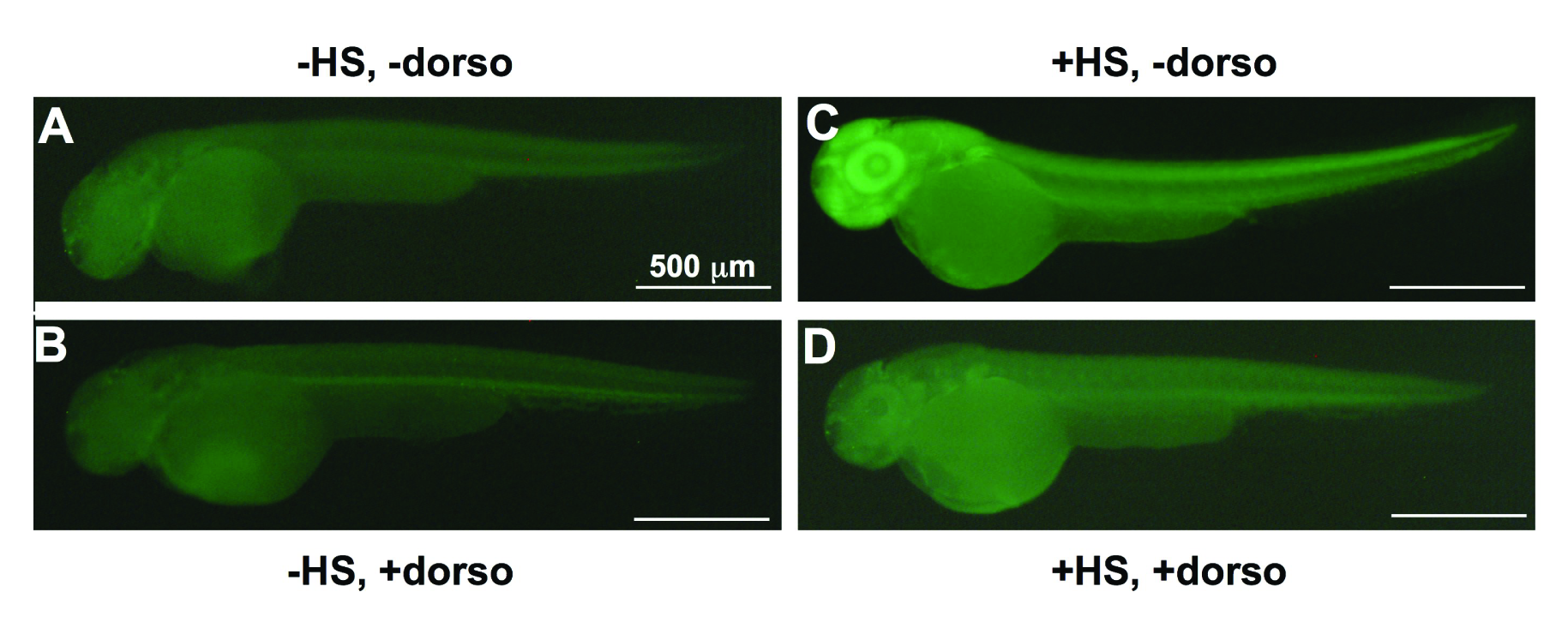

Supplement: Figure S1 — Treatment with dorsomorphin decreases BMP2b-induced phospho-smad1,5,8 staining in zebrafish embryos. Tg(hsp70:bmp2b) embryos were fixed at 55 hpf for immunohistochemical staining for phospho-smad1,5,8 following (A) no heat shock and no chemical treatment (−HS, −dorso), (B) no heat shock, but treatment with dorsomorphin (−HS, +dorso), (C) heat shock and no chemical treatment (+HS, −dorso), (D) heat shock and treatment with dorsomorphin (+HS, +dorso), representative embryos lateral view. Heat shock was performed at 48 hpf. Dorsomorphin treatment was performed from 28–55 hpf at a concentration of 40 µM. For enhanced sensitivity, a fluorescently-labeled secondary antibody was used (Alexa Fluor® 488 goat anti-rabbit IgG, Invitrogen, #A-11008). Embryos were illuminated with an X-cite Series 120 PC microscope lamp (Exfo Life Sciences and Industrial Division, Quebec, Canada) and emitted light was filtered with a green fluorescent protein (GFP) filter set. N = 15–22 embryos per group. (2.47 MB TIF) [file pone.0014553.s003.tif]

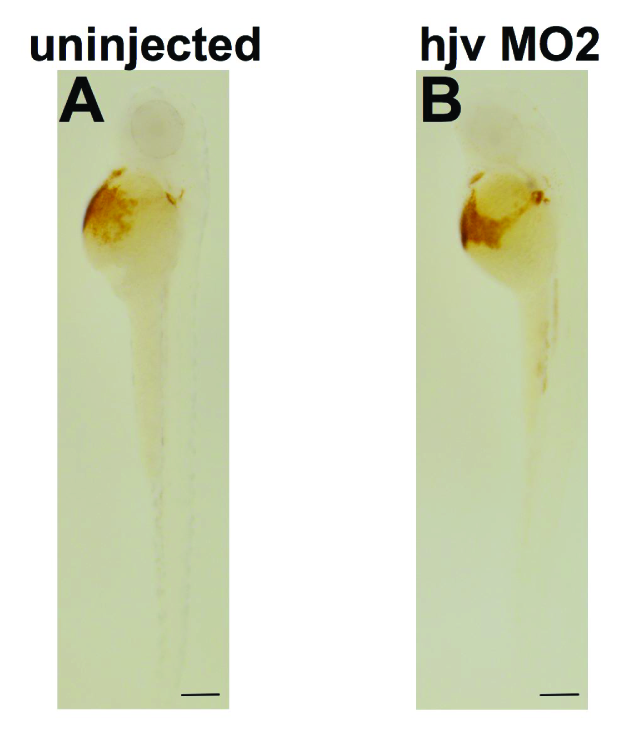

Supplement: Figure S2 — Knock down of hjv fails to produce anemia in zebrafish embryos. O-dianisidine staining for hemoglobin in embryos at 50 hpf, which were either uninjected (A) or injected with hjv MO2 (B) (lateral view). N = 42 embryos per group. (0.87 MB TIF) [file pone.0014553.s004.tif]

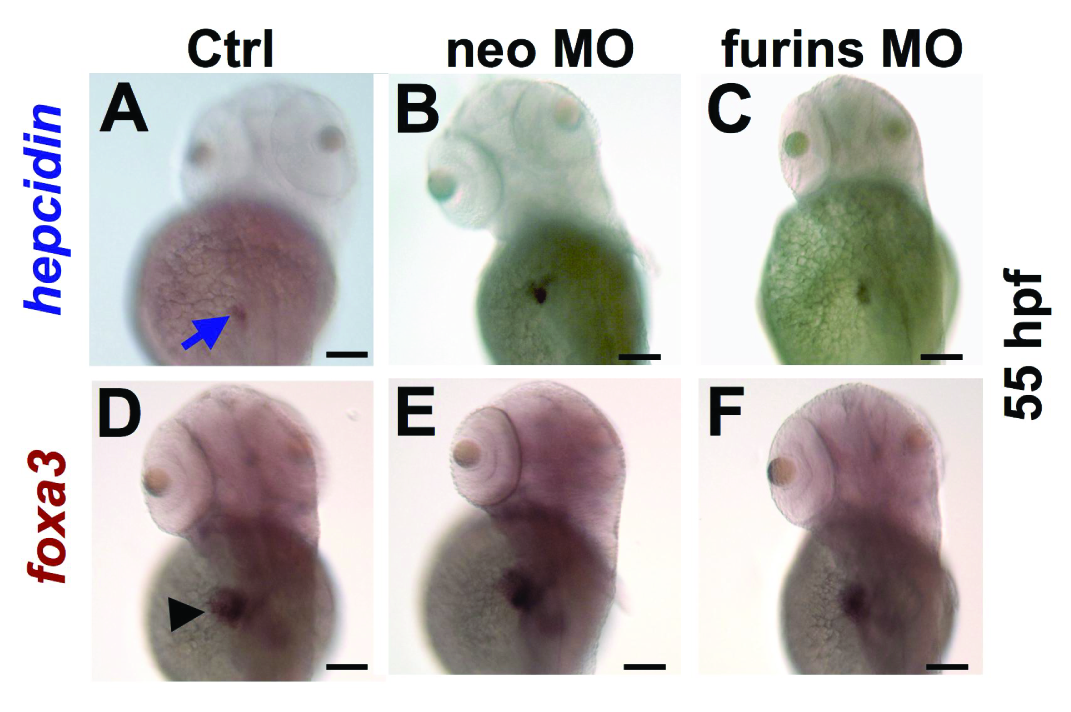

Supplement: Figure S3 — Knock down of hjv interacting proteins, neogenin or furin, fails to decrease hepcidin expression. Whole mount in situ hybridization for hepcidin (A–C, blue arrow) and foxa3 (D–F, black arrowhead) in uninjected embryos (A,D), compared to embryos injected with neogenin MO (B,E) or morpholinos directed against both zebrafish furins (furina and furinb) (C,F), dorsolateral view. N = 20 embryos per group. (0.98 MB TIF) [file pone.0014553.s005.tif]

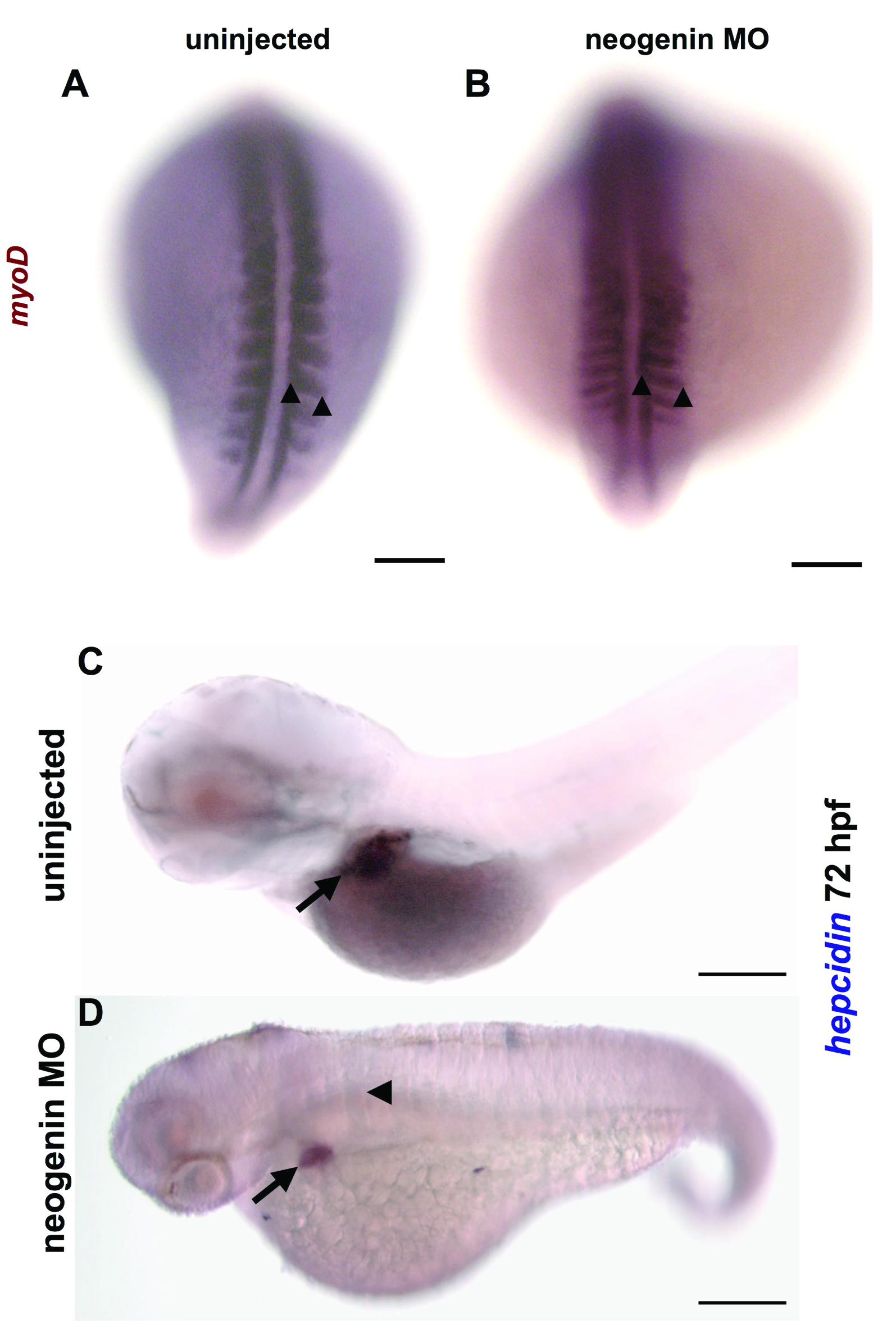

Supplement: Figure S4 — Neogenin knockdown reproduced the reported defect in somitogenesis associated with neogenin deficiency. A,B. Whole mount in situ hybridization for myoD to stain the somites in uninjected (A) and neogenin morphants (B) at the 20 somites' stage of development (dorsal view) confirmed that injection of the neogenin morpholino at 0.15 mM produced elongation of the somites, manifest by increased distance between the two arrowheads. This is characteristic of the neogenin deficient phenotype, as described by [4]. Scale bar represents 100 microns. C,D. Whole mount in situ hybridization for hepcidin at 72 hpf in uninjected control embryos (C) and neogenin morphants (D) (lateral view) revealed a shortened body axis with a curved tail and flattened somites (arrowhead) in the neogenin morphants. Hepcidin expression is present in the liver (arrow) of the neogenin morphant, although the expression domain of hepcidin is smaller than in the uninjected control. Scale bar represents 200 microns. N = 20 embryos per group. Embryos were photographed at 100x magnification with a an Axio Imager 1 compound microscope (Carl Zeiss MicroImaging, Inc., Thornwood, NY) and an AxioCam ICc1 digital camera (Carl Zeiss MicroImaging, Inc.) (A,B) or a BX51 compound microscope (Olympus, Center Valley, PA) and a Q-capture 5 digital camera (QImaging, Surrey, BC, Canada) (C,D). (3.73 MB TIF) [file pone.0014553.s006.tif]

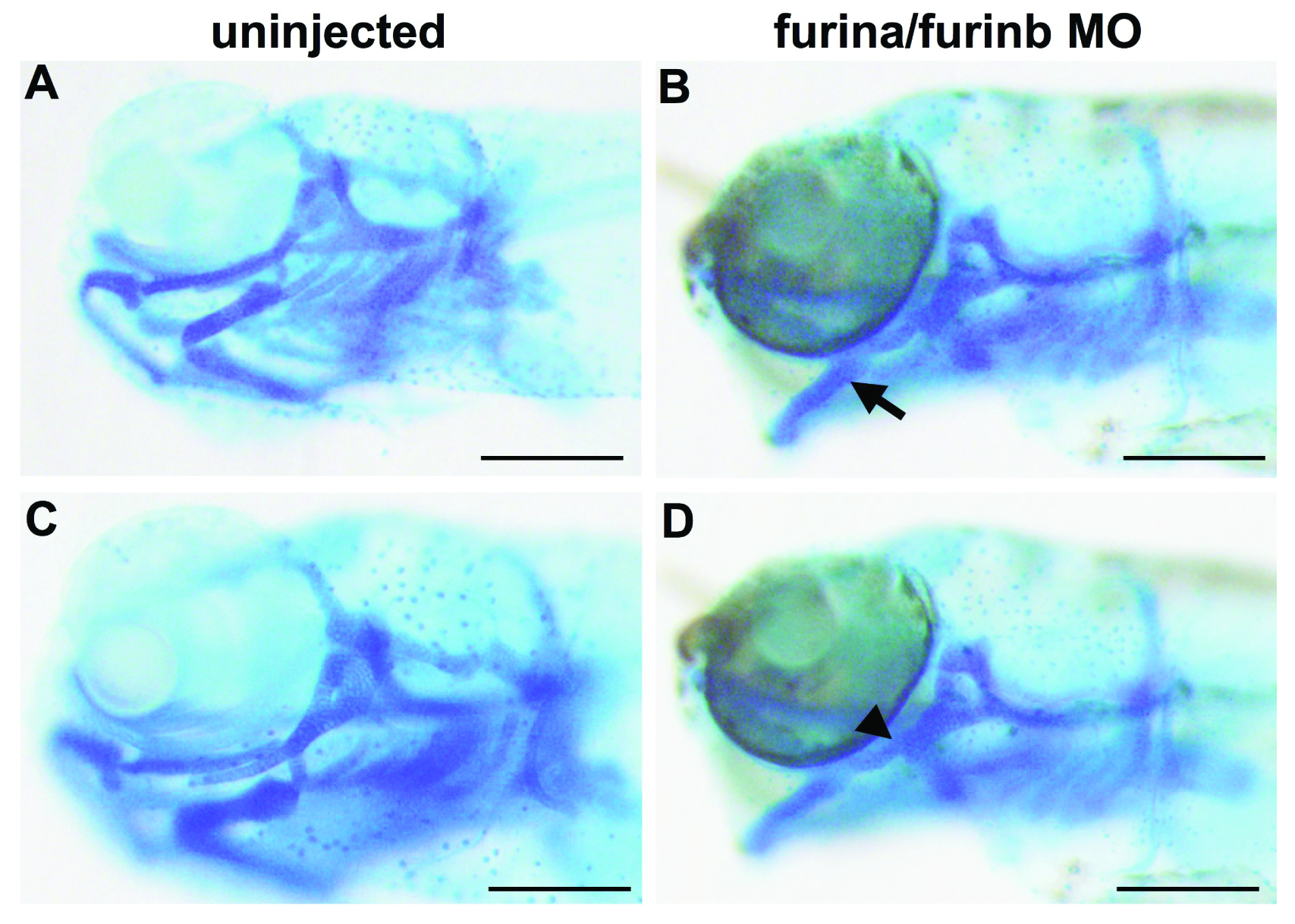

Supplement: Figure S5 — Whole mount Alcian blue staining for cartilage in zebrafish embryos at 5 days post-fertilization confirms a branchial arch phenotype in furin morphants. Dorsolateral view of the head of an uninjected control embryo (A) and an embryo injected with morpholinos to knock down furina and furinb (B) reveals an open mouth phenotype (arrow in B) in the furina/furinb morphant. Lateral view of an uninjected control (C) and a furina/furinb morphant showing the fused cartilage elements (arrowhead in D) characteristic of furin morphants. N = 20 embryos per group. (3.46 MB TIF) [file pone.0014553.s007.tif]

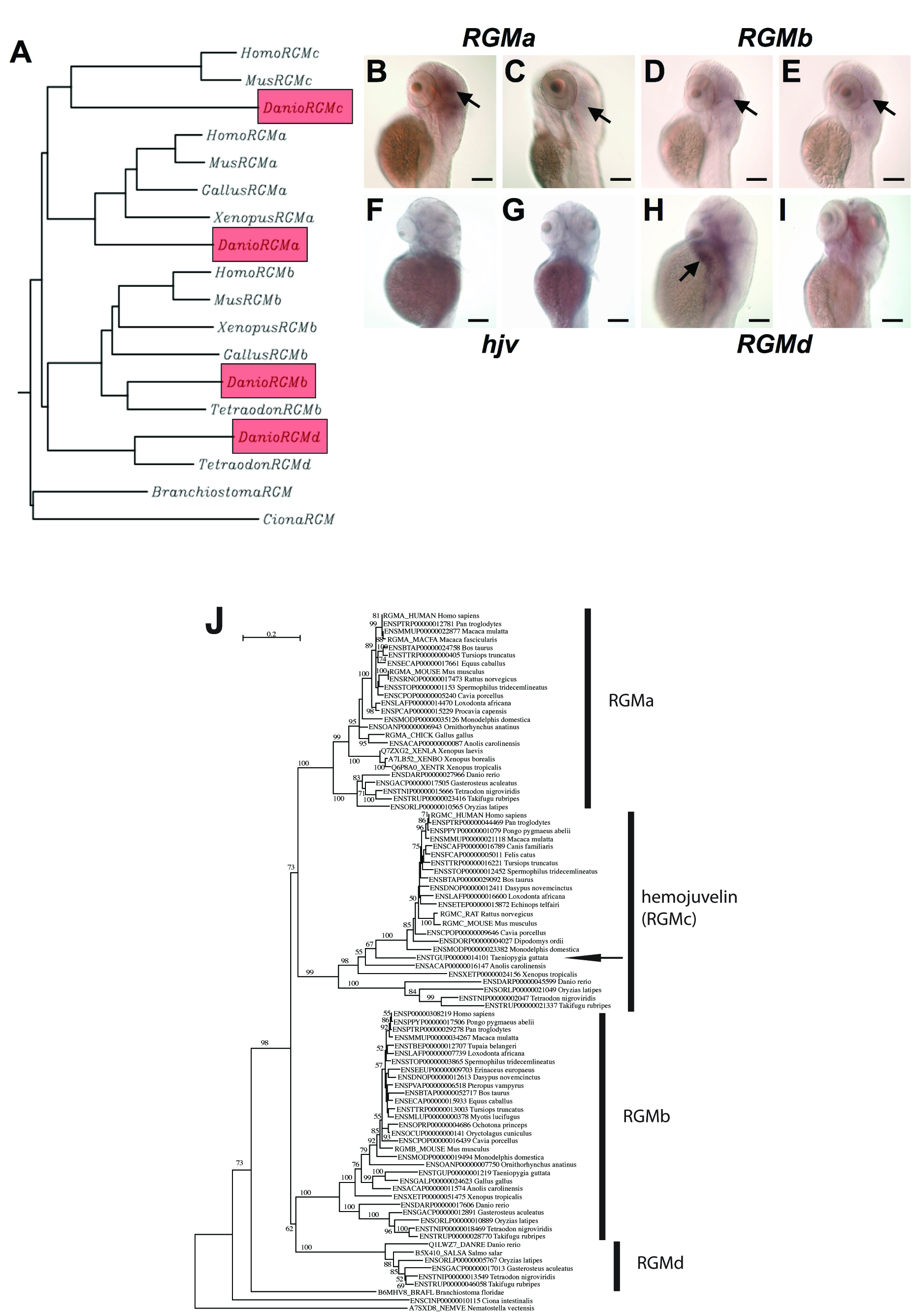

Supplement: Figure S6 — Phylogeny and expression of zebrafish RGM's. Phylogenetic tree (A) of hjv and repulsive guidance molecule genes (RGM's) in chordates. The four zebrafish RGM paralogs are highlighted in red. Hjv is also known as RGMc. B–I. Whole mount in situ hybridization of zebrafish embryos, dorsolateral views, at 50 hpf (B,D,F,H) and 72 hpf (C,E,G,I), for RGMa (B,C), RGMb (D,E), hjv (F,G), and RGMd (H,I) revealed that none of the RGM genes are detectable in the developing liver. Strong staining was detected in the mid and hindbrain for RGMa at 50 hpf (B) and 72 hpf (C, black arrows). At 50 hpf (D) and 72 hpf (E), RGMb is faintly expressed in the mid and hindbrain (black arrows). At 50 and 72 hpf, hemojuvelin is no longer detected in the developing embryo by in situ hybridization (F,G). At 50 hpf, RGMd transcripts were detected in the pharyngeal arches (H, black arrow). RGMd expression was no longer detected at 72 hpf (I). N = 20 embryos per group. (J) Phylogenetic tree of the RGM gene family constructed with all available vertebrate sequences. Note that hjv is expressed in a wide range of mammals, fish, and in Xenopus. We have identified hjv in the genome of a bird, the zebra finch (arrow), for the first time. RGMd has only been identified in fish. To generate the tree shown, we downloaded the protein sequences of the RGM gene families defined in the Ensembl database version 52 (as of December 2008) (<http://www.ensembl.org/>), which includes the hjv sequences. In addition to the Ensembl data, which also includes the Uniprot database (<http://www.uniprot.org/>), we also screened the NCBI database (<http://www.ncbi.nlm.nih.gov/>). Alignments were generated using ClustalW and Muscle[5], [6], followed by manual refinement using SeaView[7] to remove redundant and improperly annotated sequences. Phylogenetic tree reconstruction was carried out using the maximum likelihood (ML) method. Of note, the neighbor-joining (NJ) method[7] gives the same basal node topology. For ML analyses, rob [file pone.0014553.s008.tif]

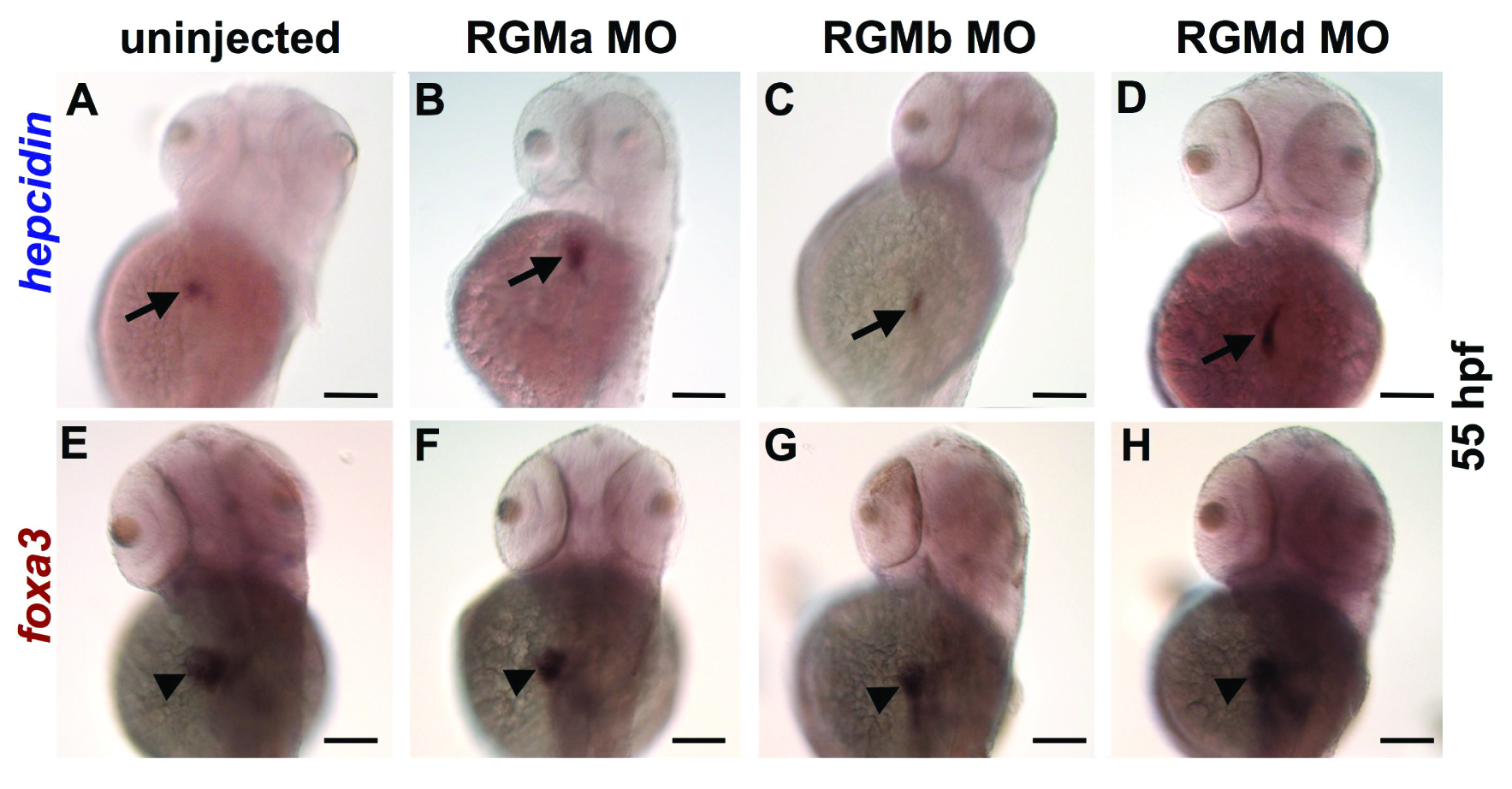

Supplement: Figure S7 — Effect of morpholino knockdown of RGM genes at 55 hpf. Whole mount in situ hybridization for hepcidin (A–D) or foxa3 (E–H), dorsolateral views. Compared to uninjected controls (A), knockdown of RGMa (B), RGMb (C), or RGMd (D) failed to inhibit hepcidin expression (arrow). E–H. Expression of foxa3 in the liver (arrowhead) revealed a slight reduction of liver size in the morphants (F–H) compared to control (E). N = 20 embryos per group. (2.64 MB TIF) [file pone.0014553.s009.tif]

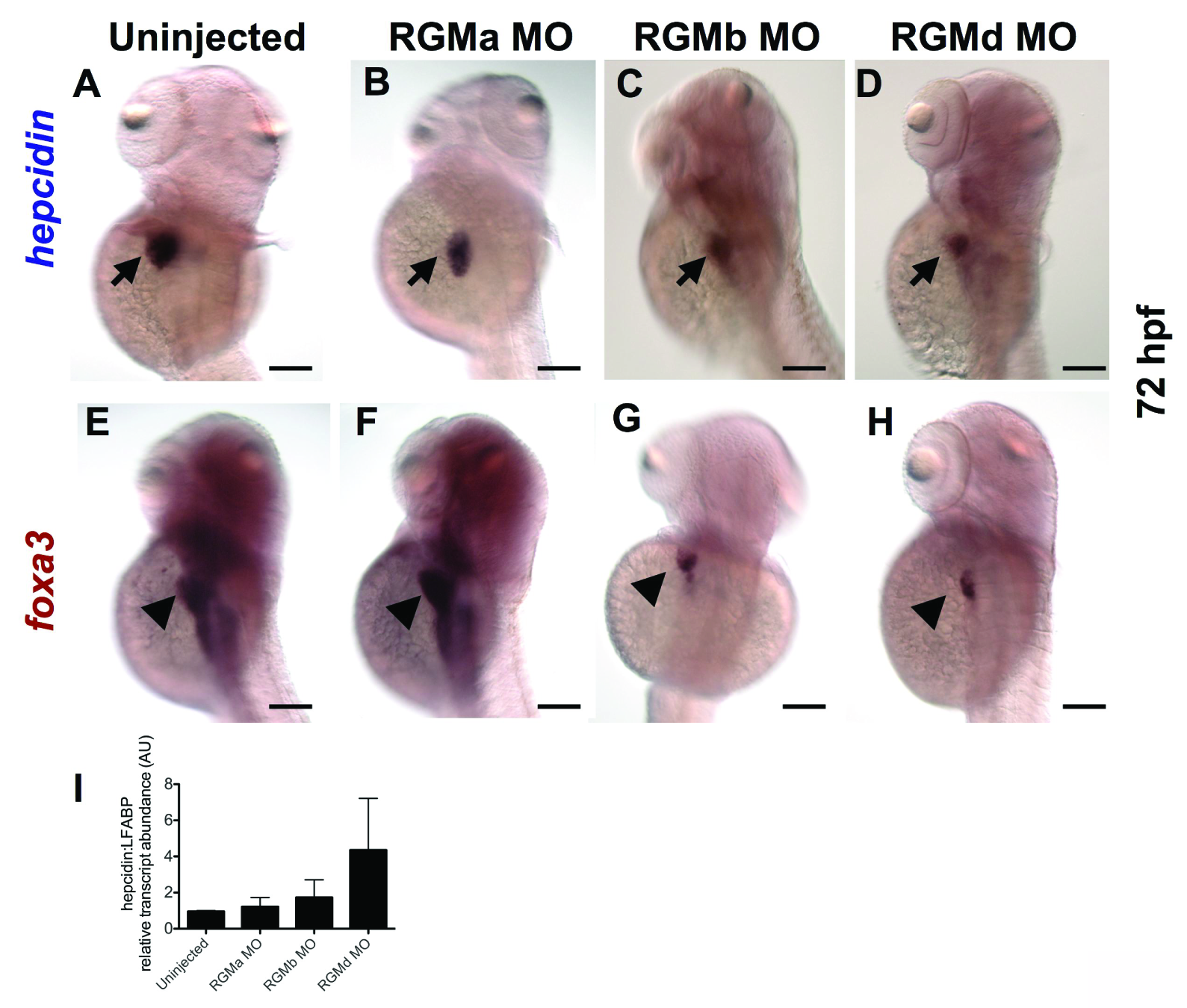

Supplement: Figure S8 — Effect of knockdown of RGM genes at 72 hpf. Whole mount in situ hybridization for hepcidin (A–D) or foxa3 (E–H), dorsolateral views. Compared to uninjected controls (A), knockdown of RGMa (B), RGMb (C), or RGMd (D) failed to inhibit hepcidin expression. E–H. Expression of foxa3 in the liver revealed a significant reduction of liver size in the RGMb and RGMd morphants (G, H). N = 20 embryos per group. I. Quantitative real-time RT-PCR revealed no significant decrease in hepcidin transcript levels relative to liver fatty acid binding protein (LFABP). N = 3 pools of embryos per group. Data shown are means + SE. (2.43 MB TIF) [file pone.0014553.s010.tif]

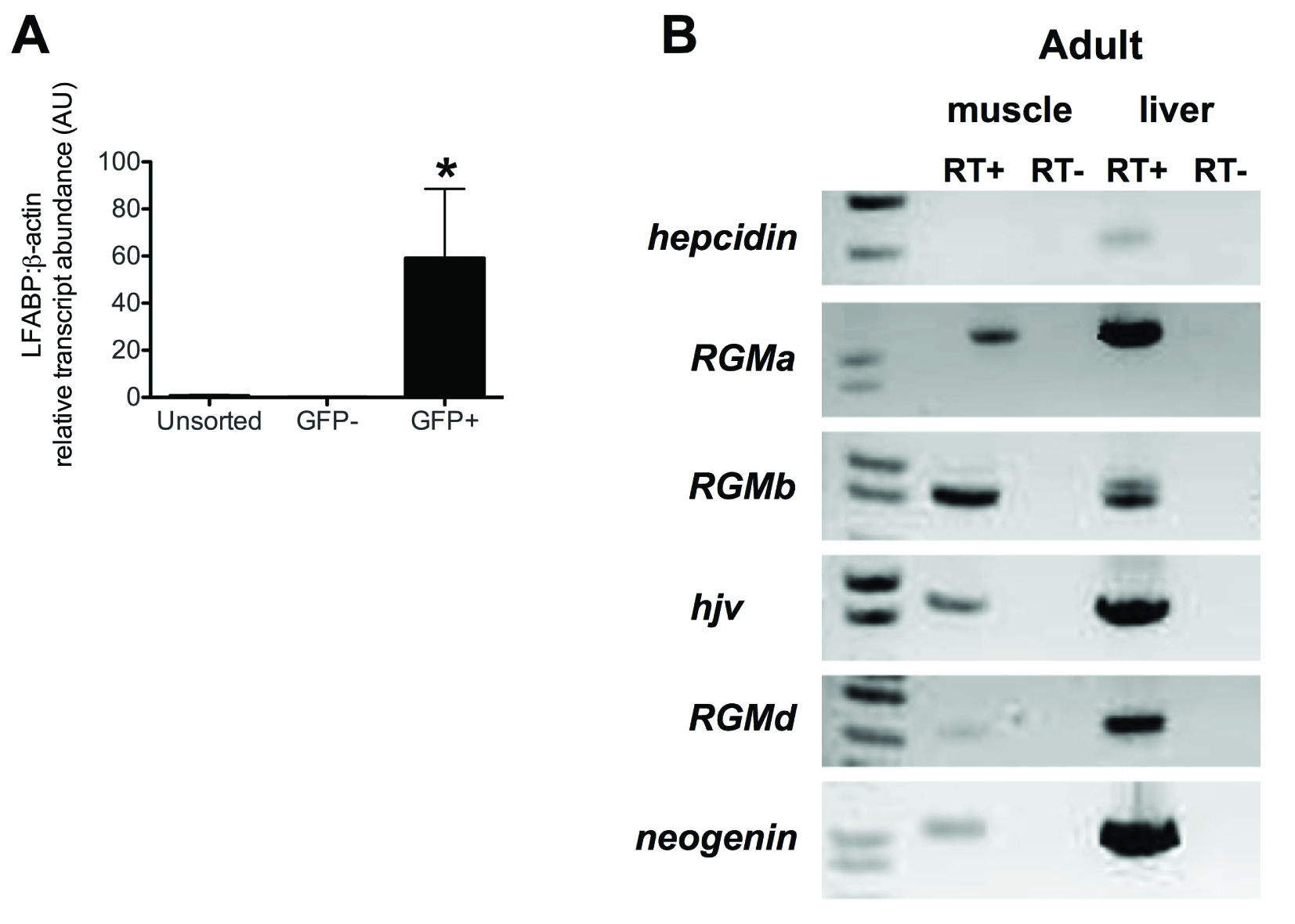

Supplement: Figure S9 — Additional expression data for zebrafish embryonic hepatocytes and zebrafish adult tissues. A. Quantitative real-time RT-PCR to assess transcript levels of LFABP (liver fatty acid binding protein) relative to β-actin in hepatocytes sorted from pools of 80–100 transgenic zebrafish embryos at 72 hpf. N = 2 pools per group. Data shown are means +/− SE. * indicates p<0.05 compared to unsorted. B. Semiquantitative RT-PCR for hepcidin, RGMa, RGMb, hjv, RGMd, and neogenin performed with RNA from adult zebrafish liver and skeletal muscle. Hepcidin expression was detected in the adult liver, but not in adult skeletal muscle. All RGM genes and neogenin were detected in the adult liver and skeletal muscle. (1.21 MB TIF) [file pone.0014553.s011.tif]

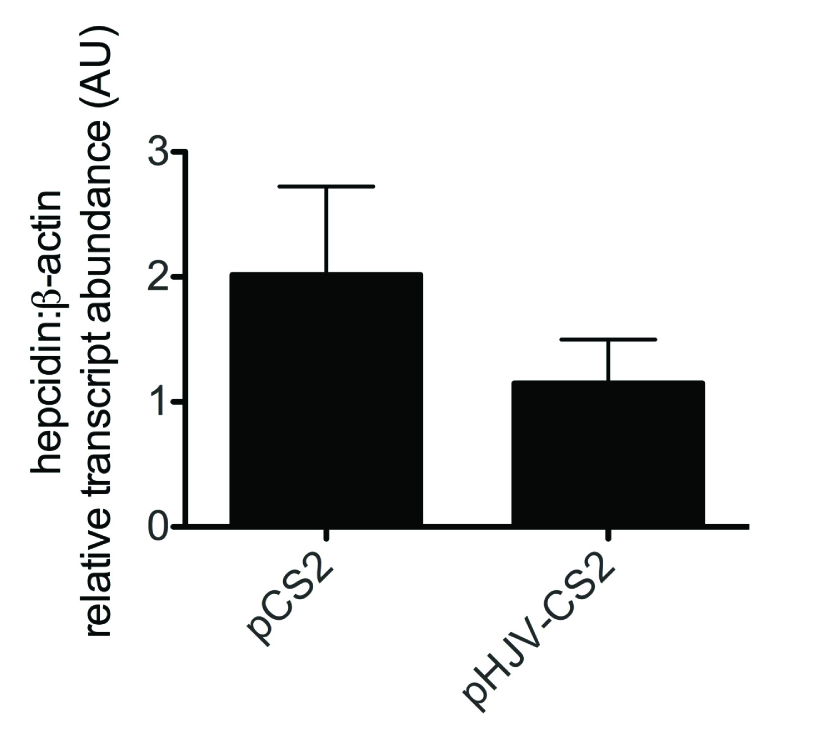

Supplement: Figure S10 — Effect of injecting zebrafish hjv cDNA in zebrafish embryos. pHjv-CS2 or pCS2 vector only (50 ng/microliter) were each injected into zebrafish embryos at the one cell stage. Quantitative real-time RT-PCR for hepcidin transcript levels normalized to β-actin expression revealed no significant increase in hepcidin expression at 55 hpf in embryos injected with pHjv-CS2 cDNA compared to pCS2 vector alone. N = 5–6 pools per group. Data shown are means +/− SE. (0.72 MB TIF) [file pone.0014553.s012.tif]

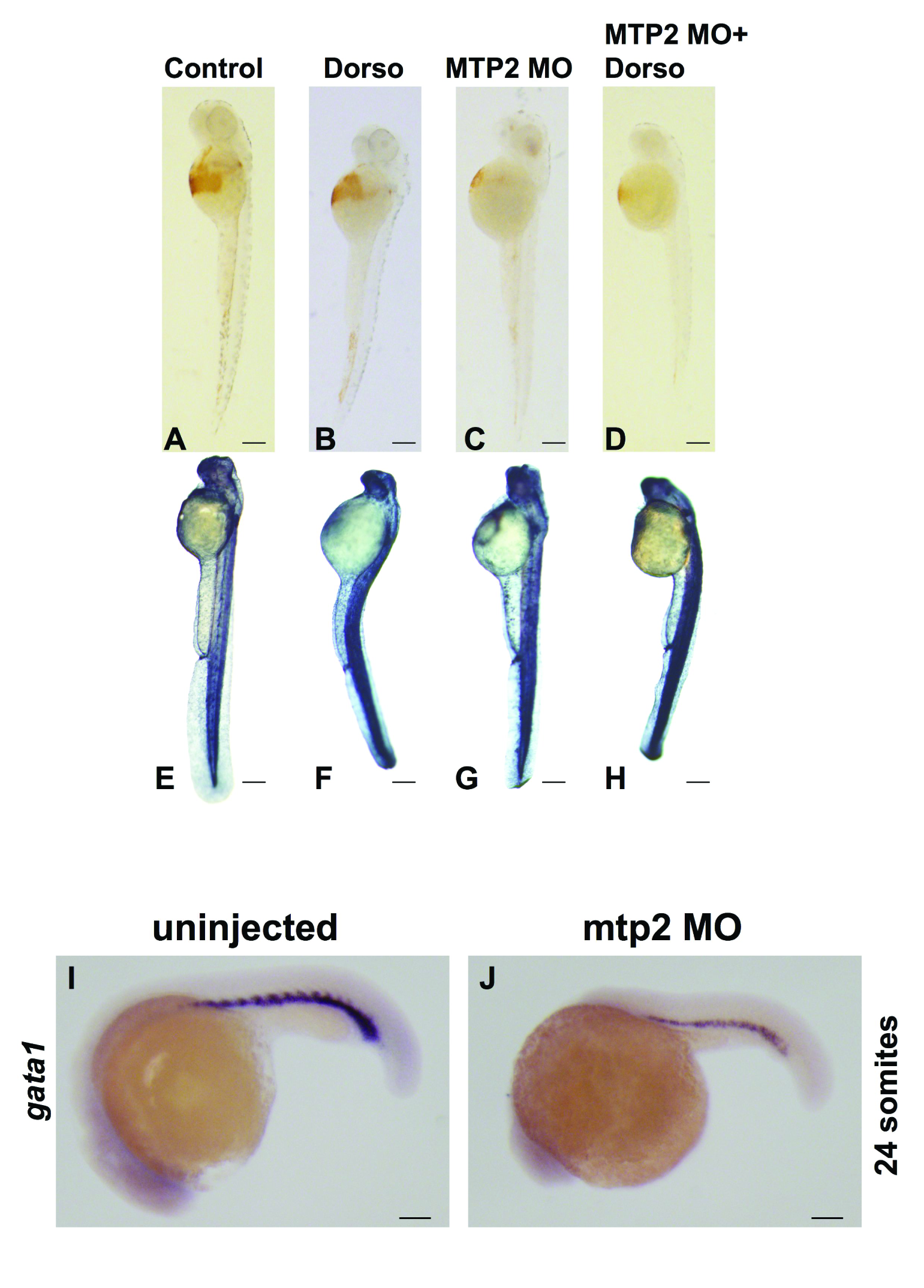

Supplement: Figure S11 — Effect of dorsomorphin on anemia and iron loading in mtp2 deficient embryos. Embryos were injected with mtp2 morpholino at the one cell stage, followed by treatment with dorsomorphin from 28 hpf until fixation for either o-dianisidine staining at 50 hpf (A–D) or whole mount nonheme iron staining at 55 hpf (E–H), lateral views. Uninjected controls (A) and embryos treated with dorsomorphin (B) exhibited normal hemoglobin staining, while mtp2 morphants (C) manifest decreased hemoglobin staining, which failed to improve when mtp2 morphants were treated with dorsomorphin (D). N = 54–99 embryos per group. Compared to uninjected controls (E), embryos treated with dorsomorphin (F), mtp2 morphants (G), or mtp2 morphants treated with dorsomorphin (H) exhibited increased iron staining in the somites, brain, and dorsal spinal cord. N = 32–45 embryos per group. (I,J) Whole mount in situ hybridization for gata1 (lateral views) when embryos have developed 24 somites, about 22 hpf, demonstrated decreased numbers of gata1-staining erythroid precursors in mtp2 morphants compared to uninjected embryos. N = 21–36 embryos per group. (3.25 MB TIF) [file pone.0014553.s013.tif]

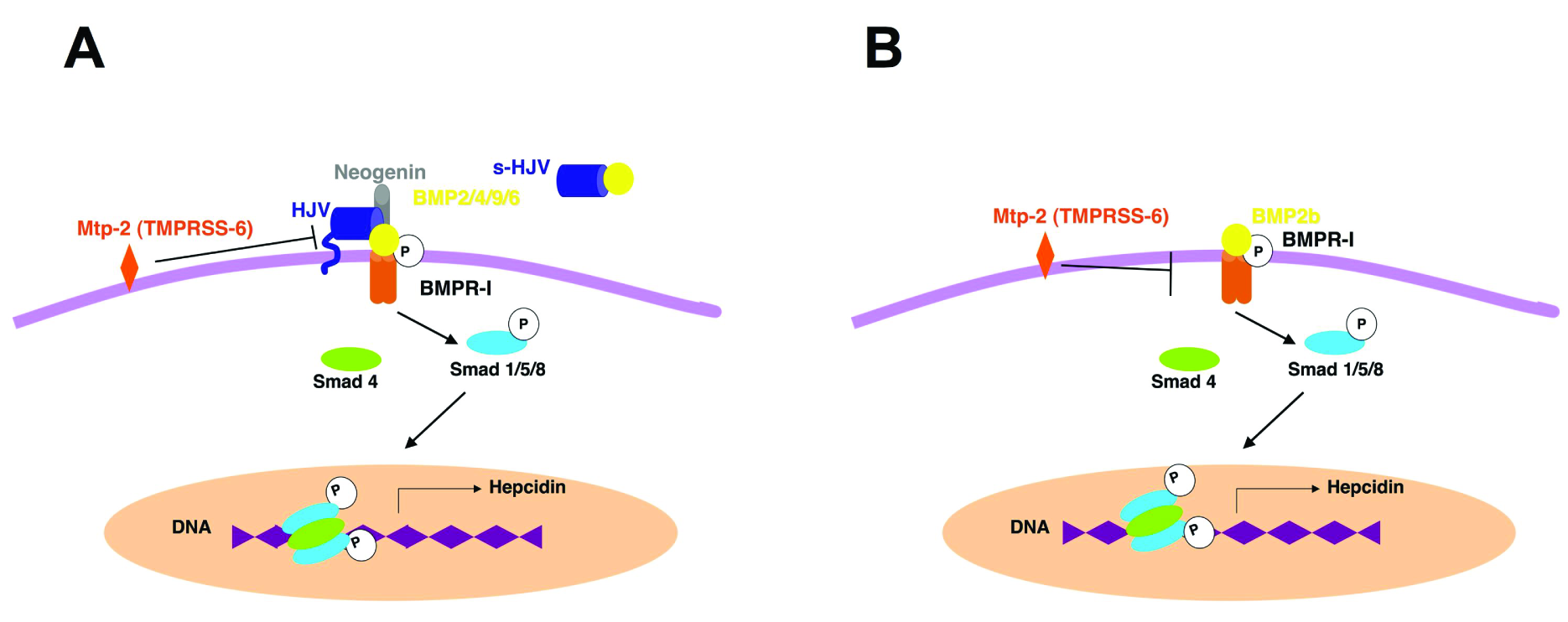

Supplement: Figure S12 — Comparison of the role of hemojuvelin in the mammalian model of hepcidin regulation with the zebrafish embryonic model. A. In the mammalian model of hepcidin regulation, which is based on in vitro studies, human patients, and post-natal animal studies[10]–[25], hjv acts as a BMP co-receptor to promote BMP signaling, which results in increased hepcidin transcription. Cleavage of membrane-bound hjv by matriptase-2 or furin results in the release of soluble hjv, which acts as a competitive inhibitor for BMP signaling. B. In the zebrafish embryonic model, which we have developed, BMP signaling promotes hepcidin transcription independent of hjv. Matriptase-2 exhibits a BMP-dependent, but hjv-independent effect on hepcidin expression. Stimulatory effects are shown by arrows. Repressive effect is shown by -|. (1.03 MB TIF) [file pone.0014553.s014.tif]
